# Large language models for preventing medication direction errors in online pharmacies

**DOI:** 10.1038/s41591-024-02933-8

**Published:** 2024-04-25

**Authors:** Cristobal Pais, Jianfeng Liu, Robert Voigt, Vin Gupta, Elizabeth Wade, Mohsen Bayati

**Affiliations:** 1 Amazon, Seattle, WA USA; 2 Department of Health Metrics Sciences, University of Washington, Seattle, WA USA; 3 Operations, Information and Technology at Graduate School of Business, Stanford University, Stanford, CA USA

**Keywords:** Health services, Technology, Medical research

## Abstract

Errors in pharmacy medication directions, such as incorrect instructions for dosage or frequency, can increase patient safety risk substantially by raising the chances of adverse drug events. This study explores how integrating domain knowledge with large language models (LLMs)—capable of sophisticated text interpretation and generation—can reduce these errors. We introduce MEDIC (medication direction copilot), a system that emulates the reasoning of pharmacists by prioritizing precise communication of core clinical components of a prescription, such as dosage and frequency. It fine-tunes a first-generation LLM using 1,000 expert-annotated and augmented directions from Amazon Pharmacy to extract the core components and assembles them into complete directions using pharmacy logic and safety guardrails. We compared MEDIC against two LLM-based benchmarks: one leveraging 1.5 million medication directions and the other using state-of-the-art LLMs. On 1,200 expert-reviewed prescriptions, the two benchmarks respectively recorded 1.51 (confidence interval (CI) 1.03, 2.31) and 4.38 (CI 3.13, 6.64) times more near-miss events—errors caught and corrected before reaching the patient—than MEDIC. Additionally, we tested MEDIC by deploying within the production system of an online pharmacy, and during this experimental period, it reduced near-miss events by 33% (CI 26%, 40%). This study shows that LLMs, with domain expertise and safeguards, improve the accuracy and efficiency of pharmacy operations.

## Abstract (web summary)

Tailored to detect and prevent potential medication direction errors in a digital pharmacy data processing pipeline, a large language model is shown to increase efficiency and decrease burden for technicians and pharmacists in a prospective application.

## Main

Medication errors, constituting a major category of medical errors, are defined as preventable mistakes that can occur at any stage of the medication-use process, including prescribing, dispensing and administering medications. These errors result in at least 1.5 million preventable adverse drug events each year in the USA and incur nearly US$3.5 billion in annual costs^1,2^. Recent studies suggest these figures may be considerably higher^3^. Although not every medication error results in harm, approximately 1% lead to adverse consequences^4^. Notably, in 1993, medication errors were implicated in about 7,000 deaths, with numerous instances of unreported adverse events and complications^3,5–9^. While over 75% of medication errors are attributed to the prescribing and administration phases, errors in pharmacies are both common and costly^6,10–12^. A national observational study in the USA reported an estimated 51.5 million dispensing errors annually in community pharmacies, with a meta-analysis supporting a 1.5% error rate^13–15^.

One of the leading types of medication errors is incorrect prescription directions^16,17^, stemming from various factors, including human errors such as typographical mistakes, miscommunication between healthcare providers, ambiguous or incomplete data entries and the complex nature of medication management^16,18,19^. A common point at which these errors occur, whether in an online or a physical retail pharmacy, is when the prescription received from a healthcare provider is being entered into the pharmacy’s computer system. For instance, inputting a prescription direction such as ‘500 mg before procedure’ can lead to confusion, requiring patients to interpret the meaning of ‘500 mg’ in relation to their medication (such as the number of tablets to take) and an unspecified route of administration. A clearer instruction such as ‘take one tablet by mouth before procedure’ reduces such ambiguity. A critical example is the incorrect transcription of ‘take 20 mg by mouth once weekly’ as ‘take 20 mg by mouth once daily’ for methotrexate oral capsules, which could result in severe adverse effects such as pancytopenia and even death^20^. The introduction of electronic health records (EHRs) adds complexity to medication direction accuracy. EHRs, while structuring data entry, also permit free-text fields for prescriptions, creating inconsistencies and potential for errors. This challenge is further exacerbated by diverse, nonstandard style guidelines used across various organizations and countries, each aligning with their operational needs^21,22^. These issues are prevalent in both online and physical pharmacy settings, underscoring the pressing need for innovative solutions to improve the accuracy of medication directions and, consequently, enhance patient safety.

**Fig. 1:**
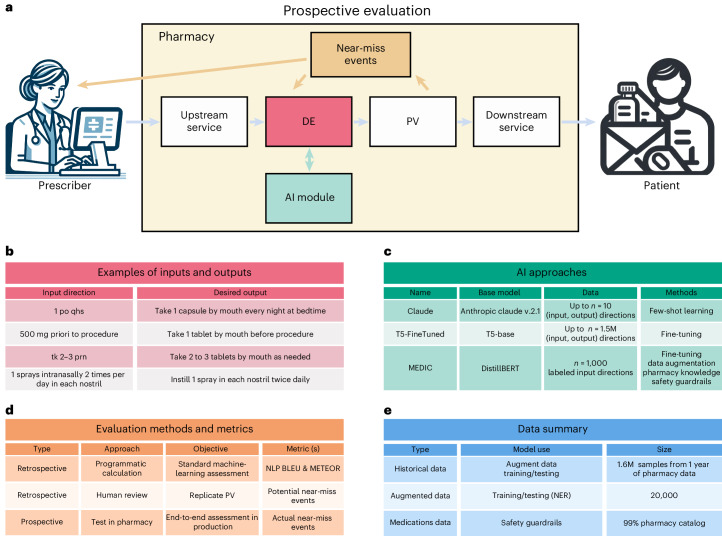
High-level overview of the study and pharmacy workflow. **a**, Schematic of the pharmacy workflow used, highlighting the occurrence of near-miss events, the primary metric of the prospective evaluation. **b**, Examples of pairs of prescriber medications directions and their corresponding, pharmacist verification (PV) equivalents. This process occurs in the DE technician step as highlighted in the previous panel. **c**, Different LLM-based strategies were used to generate pharmacist-approved medication directions from prescriber directions, highlighting their corresponding data requirements and training methodology. **d**, Types of evaluation and metric utilized to assess the performance of each AI approach. **e**, Data description and how the data were used in the study to train and evaluate the different AI approaches.

**Fig. 2:**
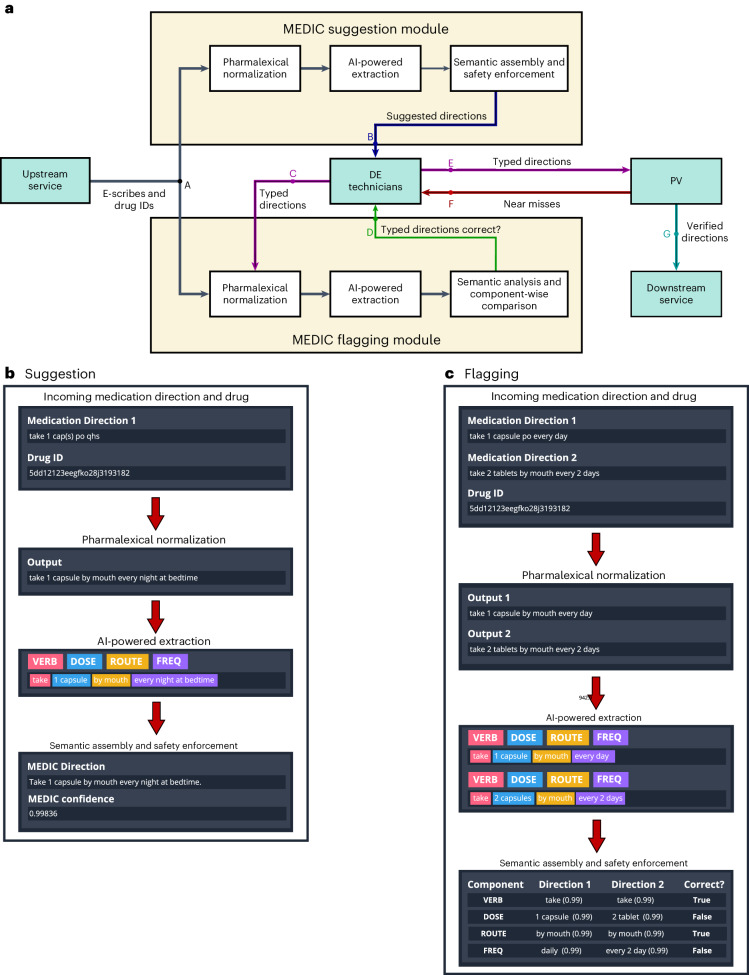
Prescription processing workflow and a high-level overview of MEDIC. **a**, Integration of the MEDIC system within the prescription processing workflow. Flow A,B, upon a DE opening a new prescription, the suggestion module activates automatically, offering proposed directions within the DE user interface. Flow C,D, each time a DE types or edits directions, the flagging module initiates, displaying flagging results in the DE user interface. Flow E, should the entered direction be deemed accurate, it advances to pharmacist verification (PV). Flow F, detected errors in the entered direction are sent back by the pharmacists for rectification. Flow G, after verification, the typed direction moves to fulfillment. **b**, Workflow of the suggestion function. Incoming medication directions from the prescriber and the associated internal drug ID serve as primary inputs. Raw directions undergo processing in pharmalexical normalization, key components are identified in AI-powered extraction and finally, directions are assembled and undergo safety checks in semantic assembly and safety enforcement. **c**, Workflow of the flagging function. Direction pairs and their associated drug IDs are primary inputs. Both sets of directions traverse the main stages of MEDIC (pharmalexical normalization and AI-powered extraction). A component-wise comparison is then conducted between the two assembled directions to identify any discrepancies.

To tackle this challenge, our paper investigates the implementation of a human-in-the-loop artificial intelligence (AI) solution, designed to enhance the standard pharmacy process, particularly the key stages of data entry (DE) and pharmacist verification (Fig. 1a). DE, a labor-intensive phase, involves pharmacy technicians transcribing prescriber directions and additional prescription details into a standardized format for efficient pharmacist review and to ensure patient understanding and safety. Figure 1b illustrates examples of these transcribed directions. In the pharmacist-verification phase, pharmacists meticulously review all information processed in the DE phase for both accuracy and potential drug interactions across the patient’s profile. This phase occasionally identifies near-miss events, defined as events where errors are caught and re-routed for correction before reaching the patient^23^, thereby preventing potential harm, as shown in Figs. 1a and 2a. The rate of near-misses is a crucial patient safety metric in pharmacy operations^23,24^. Driven by the growing advocacy for clinical decision support systems to enhance patient safety^25,26^, this paper focuses on developing an AI solution aimed at improving the accuracy and quality of the DE phase in processing medication directions, consequently reducing near-miss events.

Recognizing the key role of natural language processing (NLP) in interpreting medication directions, our study utilizes the capabilities of LLMs, known for their proficiency in textual data analysis^27^. While LLMs are not originally trained for generating medication directions, one can utilize established methodologies^28–33^ to adapt LLMs to generate medication directions from raw prescriber directions; however, a notable limitation of LLMs is their propensity for ‘hallucination’ or generating fabricated information with high confidence^34–36^, a critical concern for patient safety and an ongoing area of research^37,38^. We address this challenge by introducing, MEDIC, which fine-tunes first-generation LLMs using thousands of annotated prescriber directions. This method effectively extracts key clinical concepts and automatically applies safety guardrails to prevent hallucinatory outputs, enhancing the accuracy and safety of medication direction processing. Beyond rule-based checks informed by domain knowledge, the foundation of the guardrails lies in MEDIC’s initial extraction of core components of a direction, such as dose and frequency. Some of these components, for example route of administration, may be predetermined in our comprehensive medication database. The accuracy of this database ensures MEDIC is halted whenever its outputs deviate from expected self-consistency^39^ for these known components.

We also developed two benchmark applications of LLMs: one based on fine-tuning, referred to as T5-FineTuned, and the other on few-shot prompting, named Claude^40^. We then retrospectively compared MEDIC with the benchmarks on held-out medication directions data, through standard NLP metrics, the patient safety metric of near-miss rate and clinical severity assessed by pharmacists, in addition to runtime efficiency as a proxy for service level agreement in a production environment (Fig. 1d). MEDIC is then tested prospectively by deployment in the production environment of an online pharmacy (Fig. 1a).

## Results

**Extended Data Fig. 1:**
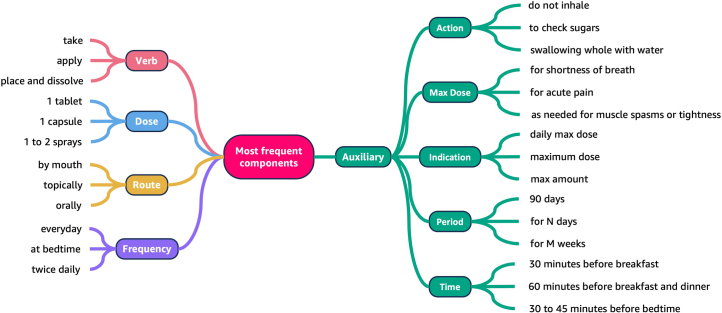
Hierarchy of components in each direction. The nine components identified by MEDIC - verb, dose, route, frequency, auxiliary actions, indications, max dose, period, and time - are represented by nodes and three examples of each component are depicted in their leaves.

Medication directions provided by healthcare providers outline how patients should take their medications, typically involving core components such as verb, dose, route, frequency and auxiliary information. For instance, the direction ‘Take one tablet by mouth once daily for pain’ includes the verb (take), dose (one tablet), route of administration (by mouth), frequency (once daily) and auxiliary information (for pain). Common examples and a detailed discussion are available in Extended Data Fig. 1 and Methods section ‘Creating MEDIC’. While most directions are single-line, conveying each component as a single piece of information, multi-line directions include multiple pieces for components such as dose and frequency and are more error prone; however, analysis of the pharmacy data available for this study reveals that over 98% of medication directions are single-line, hence the study’s focus on this predominant category.

Our training and testing data for MEDIC and the benchmarks consist of a random subsample of approximately 1.6 million single-line medication directions from a year’s worth of Amazon Pharmacy data. This dataset includes raw digital directions from prescribers and directions typed by DE technicians and verified by pharmacists. Summary statistics for this dataset are detailed in the Supplementary Fig. 1. The dataset was randomly divided into four subsets: ${{{{\mathcal{D}}}}}_{{{{\rm{H}}}}}$, ${{{{\mathcal{D}}}}}_{{{{\rm{Train}}}}}$, ${{{{\mathcal{D}}}}}_{{{{\rm{Test}}}}}$ and ${{{{\mathcal{D}}}}}_{{{{\rm{Eval}}}}}$. The ${{{{\mathcal{D}}}}}_{{{{\rm{H}}}}}$ subset, with 1,000 samples annotated by human experts for core components, was utilized to train MEDIC. The ${{{{\mathcal{D}}}}}_{{{{\rm{Eval}}}}}$ subset, including 1,200 samples, served for both NLP and human evaluations, whereas the ${{{{\mathcal{D}}}}}_{{{{\rm{Test}}}}}$ subset, with 20,000 samples, provided robustness check for NLP evaluations. To evaluate the data efficiency of MEDIC, which relies on just 1,000 samples in ${{{{\mathcal{D}}}}}_{{{{\rm{H}}}}}$, we trained a benchmark model using a considerably larger dataset—the ${{{{\mathcal{D}}}}}_{{{{\rm{Train}}}}}$ subset, comprising 1.5 million samples. Detailed explanations are available in Extended Data Table 1 and Methods. To power the safety guardrails of MEDIC, we also created a medication database, ${{{{\mathcal{D}}}}}_{{{{\rm{MedCat}}}}}$, from RxNorm^41^, OpenFDA^42^ and Amazon Pharmacy’s database, containing medication attributes and select core components essential for generating standardized directions (Methods).

The two benchmarks, T5-FineTuned and Claude, are developed through well-established methodologies using varying sample sizes from ${{{{\mathcal{D}}}}}_{{{{\rm{Train}}}}}$ as discussed in Extended Data Table 2 and Methods. Our AI module, MEDIC, uses a three-stage process, starting with a rule-based model that leverages pharmacy knowledge to format and standardize raw prescriber directions. The second stage, AI-powered extraction, and the heart of MEDIC, uses a fine-tuned DistilBERT^43^ to extract core direction components. This stage uses ${{{{\mathcal{D}}}}}_{{{{\rm{H}}}}}$ along with two synthetic datasets, ${{{{\mathcal{D}}}}}_{{{{\rm{HLA}}}}}$ and ${{{{\mathcal{D}}}}}_{{{{\rm{HLAT}}}}}$, each containing 10,000 samples, for training and validation. The final stage of MEDIC assembles medication directions using pharmacy knowledge and ${{{{\mathcal{D}}}}}_{{{{\rm{MedCat}}}}}$, depicted in Fig. 2b,c and Supplementary Table 1. It applies safety guardrails developed with pharmacists: direction generation stops if there is a conflict with ${{{{\mathcal{D}}}}}_{{{{\rm{MedCat}}}}}$ (GR1), multiple core component values (GR2), a dose without a verb (GR3), a missing frequency (GR4) or no dose with tablet/capsule form (GR5). See Methods for details.

### Retrospective NLP evaluations

**Fig. 3:**
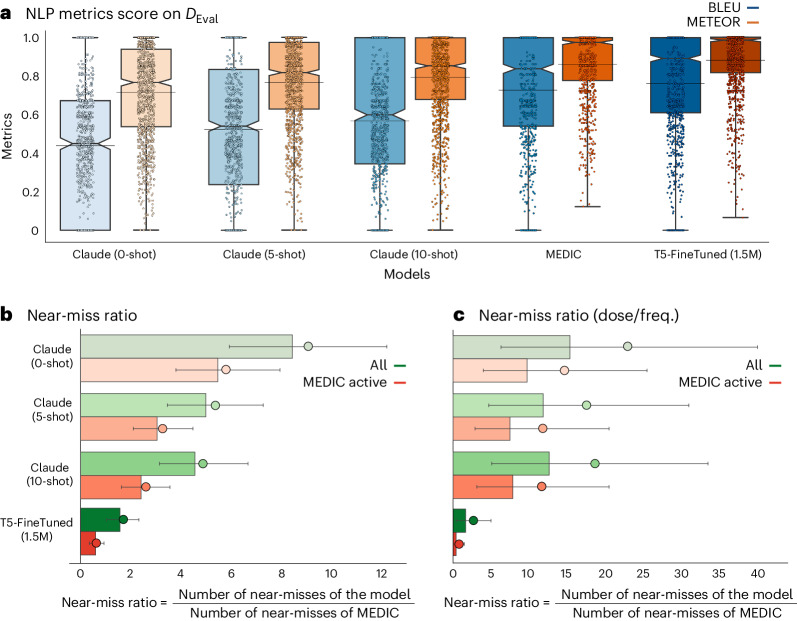
Evaluation metrics on ${{{\boldsymbol{\mathcal{D}}}}}_{{{{\bf{Eval}}}}}$ for the three AI approaches. **a**, Distribution of NLP scores BLEU and METEOR for MEDIC, T5-FineTuned (1.5M) and Claude calculated across all suggested directions (*n* = 1,200 prescriptions). Average values are indicated with an horizontal black line and median values are highlighted with a notch on each box-plot. Whiskers extend from the first and third quartiles (box limits) toward the min/max observed values for each metric and model, respectively. **b**, Comparison of ratios of all categories of possible near-miss events from a total of *n* = 1,200 prescriptions of different models with respect to MEDIC, with their 95% percentile intervals represented by black lines obtained via bootstrap^59^ to account for the ratios’ skewed distribution, with their centers representing the median values. **c**, Comparison of ratios highlighting near-misses related to incorrect dosage or frequency from a total of *n* = 1,200 prescriptions, which carry an elevated risk of patient harm, with their 95% percentile intervals represented by black lines obtained via bootstrap to account for the ratios’ skewed distribution, with their centers representing the median values.

Figure 3a shows results from 1,200 prescriptions in ${{{{\mathcal{D}}}}}_{{{{\rm{Eval}}}}}$, where T5-FineTuned (1.5M)—fine-tuned with 1.5 million pairs of prescriber medication directions and their corresponding, pharmacist-verified equivalents—and MEDIC closely match in BLEU and METEOR metrics, with a slight edge for T5-FineTuned (1.5M). It also demonstrates that increasing examples of such paired directions in few-shot learning with Claude improves performance, but still lags substantially behind T5-FineTuned (1.5M) and MEDIC. The evaluation of 20,000 prescriptions in ${{{{\mathcal{D}}}}}_{{{{\rm{Test}}}}}$, as detailed in Supplementary Fig. 4, not only supports our findings from ${{{{\mathcal{D}}}}}_{{{{\rm{Eval}}}}}$ but also highlights the importance of training data volume in pharmacy context, challenging the idea that minimal data suffice for optimal LLM fine-tuning^44–46^. Notably, T5-FineTuned demonstrates underperformance against a rule-based model (defined in the Methods section ‘Evaluations’) when fine-tuned with only 100 samples; however, its performance enhances with 1,000 samples and surpasses Claude (ten-shot) when fine-tuned with a larger dataset of 10,000 samples. With 1.5 million samples, it even slightly outperforms MEDIC.

#### Limitations of NLP metrics

Despite the utility of BLEU and METEOR scores in comparing large datasets like ${{{{\mathcal{D}}}}}_{{{{\rm{Test}}}}}$, they have limitations^47^, particularly in capturing clinical severity. High scores may not reflect the safety or accuracy of medication directions; for example, subtle deviations in verbs (for example, swapping ‘take’ for ‘dissolve’) or frequencies (for example, mistaking ‘every 4 hours’ with ‘every 12 hours’), can lead to substantial clinical risks. Supplementary Table 3 presents instances where suggestions, while metrically accurate, are clinically incorrect, emphasizing the necessity of detailed human evaluation, which we discuss in the following section.

### Retrospective human evaluation

A manual review of 1,200 ${{{{\mathcal{D}}}}}_{{{{\rm{Eval}}}}}$ samples assessed direction suggestions from MEDIC, T5-FineTuned (1.5M) and Claude versions, identifying errors that, if missed by a DE technician and passed to the pharmacist-verification stage, could lead to near-miss events. The outcomes of this evaluation are depicted in Fig. 3b. Owing to confidentiality, the near-miss ratios relative to MEDIC are displayed, with separate presentations for the overall 1,200 samples (All) and for those where MEDIC’s guardrails permitted direction generation (MEDIC Active). Moreover, Fig. 3c specifically addresses near-misses related to incorrect dosage or frequency, which carry an elevated risk of patient harm depending on the medication due to the potential for under/overdosing.

Results from Fig. 3b align with our NLP-based evaluations, showing that all Claude versions have the potential of generating more near-misses than MEDIC and T5-FineTuned (1.5M). Notably, the best variant of Claude records 4.38 times (CI 3.13, 6.64) more near-misses than MEDIC. While incrementing the number of shots (examples) in Claude enhances its performance, the benefit diminishes with additional shots.

The comparison between MEDIC and T5-FineTuned (1.5M) is nuanced; across all ${{{{\mathcal{D}}}}}_{{{{\rm{Eval}}}}}$ samples, T5-FineTuned (1.5M) generates 1.51 (95% CI 1.03, 2.31) times more near-misses than MEDIC. MEDIC effectively reduces its near-miss rate by ceasing direction generation when safety guardrails are activated, covering about 80% of cases that we refer to as MEDIC Active cases (this fraction is rounded to the nearest multiple of 20% to maintain confidentiality). Focusing on MEDIC Active cases, T5-FineTuned (1.5M) outperforms MEDIC with a near-miss ratio of 0.58 (95% CI 0.33, 0.92). Nevertheless, when faced with the more intricate cases, outside of the MEDIC Active group, T5-FineTuned (1.5M), unlike MEDIC, fails to recognize its own limitations and confidently churns out directions. We found that the confidence score produced by T5-FineTuned (1.5M) is misleadingly high, rendering it unsuitable for addressing these challenging cases in which the model’s performance is subpar. This tendency toward overlooking its shortcomings results in heightened near-misses, which ultimately undermines its efficacy on the entire ${{{{\mathcal{D}}}}}_{{{{\rm{Eval}}}}}$ set.

Results in Fig. 3c mirror those in Fig. 3b but show larger confidence intervals due to fewer dose and frequency near-misses. The difference between MEDIC and T5-FineTuned (1.5M) is not statistically significant here. Nonetheless, the near-miss ratios for the Claude versions are higher compared to those in Fig. 3b, indicating heightened risks associated with Claude in near-misses due to dosage and frequency.

#### Clinical significance of near-misses

The near-misses identified during our human review were further evaluated by pharmacists to determine their clinical severity and potential harm to patients, considering the specific medications prescribed in each case. This secondary evaluation indicated that both MEDIC and T5-FineTuned (1.5M) had a minimal occurrence of clinically severe near-misses, with no statistical difference between them (*P* = 0.58); however, the best performing (ten-shot) version of Claude showed a notably higher frequency of clinically severe near-misses. Specifically, it had 5.87 (95% CI 2.1, 19.0) times more clinically severe near-misses compared to MEDIC across all cases and 4.36 (95% CI 1.44, 14.0) times more clinically relevant near-misses in MEDIC Active cases. This substantial disparity highlights the marked difference in performance between Claude (ten-shot) and the other models, particularly in terms of the risk of serious harm to patients.

Errors by Claude (ten-shot) encompass various critical mistakes that could substantially endanger patient safety. Notably, numerous instances included dosage inaccuracies that risked underdosing or overdosing, particularly critical in medications requiring precise dosing. Furthermore, there were several cases of incorrect frequency instructions, raising the potential for dangerously high medication levels. A glaring example involved adding an extra insulin administration at bedtime, which could lead to a lethal overdose, especially if the patient is also on long-acting insulin at that time. Additionally, the omission of specific timing instructions in some cases heightened the risk of harmful drug interactions. Moreover, we observed errors concerning the route of administration, which could either impact the efficacy of the drug absorption or, in certain situations, cause direct harm to the patient.

#### Investigation of MEDIC’s safety guardrails

**Fig. 4:**
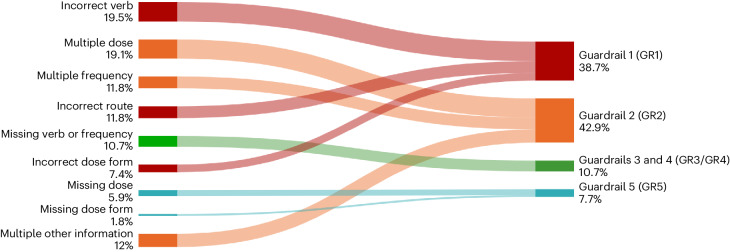
MEDIC safety guardrails triggered on human evaluation set ${{{\boldsymbol{\mathcal{D}}}}}_{{{{\bf{Eval}}}}}$. Safety guardrails trigger reasons and their percentage over the total number of blocked suggestions (left). Guardrails mapping from trigger reasons and the total percentage of blocked suggestions falling into the specific guardrail (right).

Figure 4 provides a detailed analysis of the safety guardrails in MEDIC, which proactively prevent the AI module from generating directions for certain cases. This analysis reveals the frequency and types of triggers for each guardrail, highlighting the critical role of the medication catalog data, ${{{{\mathcal{D}}}}}_{{{{\rm{MedCat}}}}}$, described above. It also showcases the various cases where incoming direction components, such as verb, route and dose form, result in halted suggestions. Furthermore, the figure illustrates the proportion of instances where multiple values for core components or missing essential information lead to guardrail activation.

#### MEDIC’s secondary function: flagging technician errors

**Fig. 5:**
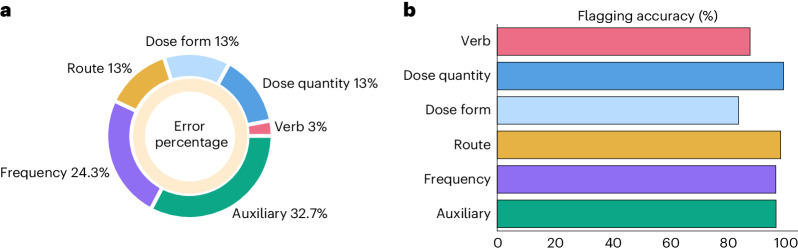
Offline flagging model performance in detecting different direction errors. **a**, Error percentage distribution across all relevant components of the medication directions. **b**, MEDIC flagging model accuracy for each component.

AI-powered extraction the second stage of MEDIC, designed to extract core components from prescriber directions, offers an additional layer of safety. This is achieved by comparing the extracted components with those derived from the DE phase outputs. This process effectively flags, in real time, instances where the prescriber and DE directions diverge from a clinical standpoint (refer to Fig. 2c and Methods for details). To evaluate the efficacy of MEDIC’s flagging function, we tested it on historically validated near-miss events returned for corrections by pharmacists. These encompassed six error categories, with auxiliary errors being most prevalent, followed by dose and frequency errors. Detailed error distributions are outlined in Fig. 5a. MEDIC successfully flagged 95.1% of these errors, and was especially effective for errors related to dose quantity, route, frequency and auxiliary (Fig. 5b); however, it was less effective in detecting verb and dose form errors primarily due to incomplete data in ${{{{\mathcal{D}}}}}_{{{{\rm{MedCat}}}}}$. Future enhancements could involve integrating additional data sources such as notes, dispensing history and customer details to refine error detection.

### Qualitative study of LLMs hallucinations and comparison with ChatGPT4 and Gemini Pro

To further illustrate instances of LLM hallucinations, we analyzed cases where T5-FineTuned (1.5M) and Claude were unable to restrict their suggestions within the confines of an automated online pharmacy system. Extended Data Table 3, adhering to Amazon’s confidentiality policies, presents synthesized yet realistic examples of prescriber directions and outputs from MEDIC and T5-FineTuned (1.5M). These examples reveal T5-FineTuned (1.5M) frequently missing key prescriber details such as frequency, route and medication type, especially in examples 1–3. When faced with incomplete information, the model introduces errors such as incorrect dose forms and verbs (examples 4–8) and in a concerning case, example 9, it entirely fabricates an unrelated direction, highlighting potential risks.

We assessed whether ChatGPT4 and Gemini Pro (Google Bard’s upgrade on 6 December 2023)^48^, accessed on 29 December 2023, could address the limitations identified in T5-FineTuned (1.5M) and Claude. For fair comparison, we used the same synthesized prescriber medication directions for these models as for Claude (zero-shot), as seen in Extended Data Table 3. Results from Extended Data Table 4 show that ChatGPT4 and Gemini Pro’s outputs are structurally and stylistically similar to Claude, and somewhat to T5-FineTuned (1.5M), even without fine-tuning and just using zero-shot prompts. This underscores the inherent strengths of modern LLMs but also highlights the necessity for additional context or refined fine-tuning to meet healthcare safety standards. Key observations include: in examples 1 and 5, all models inaccurately imply a tablet or capsule form; Gemini Pro erroneously adds dose or instructions in examples 2 and 6; all models assume ‘intramuscular’ as the administration route in example 3 and erroneously add ‘by mouth’ in example 7; in example 8, they inappropriately use the verb ‘take’; and in example 4, Claude adds extra instructions. Most concerning, in examples 9 and 10, all or two models fabricate or misinterpret dosage information, respectively. Sensitivity analysis, involving enriched prompts with specific medication details from ${{{{\mathcal{D}}}}}_{{{{\rm{MedCat}}}}}$ (elaborated in Methods), led to improved outputs from all models, particularly for examples 4 and 5. Nonetheless, complex scenarios such as example 9 remained challenging.

Although LLM-based approaches such as T5-FineTuned, Claude, ChatGPT4 or Gemini Pro offer simplicity in deployment, our research reveals their limitations in critical areas such as pharmacy, even with extensive fine-tuning or few-shot prompting. Despite potential in further enhancing them with more advanced prompting techniques^32,33,49,50^, effectively managing the generative nature of these models for precise medication direction remains a hurdle. This complexity highlights the necessity for domain-specific approaches, like the safety guardrails in MEDIC, essential in healthcare for ensuring precision and safety^51^.

#### Cost and speed

As shown in Extended Data Table 2, MEDIC and T5-FineTuned showcased average execution times of 200 ms and 1 s on ${{{{\mathcal{D}}}}}_{{{{\rm{Test}}}}}$, respectively, compared to 7.6–8.2 s for Claude’s 0–10-shot versions, with similar speeds for ChatGPT4 and Gemini Pro. Unlike the latter models which have usage costs, MEDIC and T5-FineTuned are free and compatible with low-cost computing (CPU only).

### Prospective evaluation of MEDIC

The integration of MEDIC into pharmacy operations has demonstrably enhanced both operational efficiency and patient safety metrics (defined in Extended Data Table 5), in contrast to the then-active production baseline (a conventional hybrid rule-based and machine-learning-based model). Acknowledging limitations in before-and-after studies, the deployment of MEDIC during its experimental evaluation, complemented by human-in-the-loop feedback (detailed in Methods section ‘Evaluations’), yielded significant improvements. Notably, there was a 33% (95% CI 26%, 40%) reduction in near-miss events related to medication directions, an 18.3% (95% CI 17.8%, 18.9%) increase in suggestion coverage rates, a 28.5% (95% CI 28.1%, 29.0%) rise in suggestion adoption by DE technicians and a 44.3% (95% CI 43.2%, 45.4%) decrease in post-adoption edits. These edits ranged from major revisions to subtler stylistic or formatting changes before proceeding to pharmacists for final review.

MEDIC’s integration empowers technicians and pharmacists to focus on intricate, high-priority cases, thereby enhancing processing efficiency and reducing fatigue. This shift leads to a cascade of benefits: fewer errors in critical cases, faster and more-accurate prescription processing and fewer prescriptions requiring re-evaluation due to near-miss errors. This approach not only enhances safety but also reduces labor and processing costs per prescription. A formal analysis of these cost savings is an intriguing area of future research.

However, implementing AI solutions such as MEDIC in real-world environments, especially those involving human interaction, presents challenges. It demands a lengthy period of evaluation and optimization, necessitating strong collaboration with DE technicians and pharmacists. Building trust in AI-generated suggestions and incorporating ongoing feedback are essential. Discrepancies between the retrospective accuracy (95.1%) of MEDIC’s flagging function and its prospective performance highlight the complex nature of real-world applications. Factors such as unpredictable human behavior, system limitations and data quality issues in a human-in-the-loop setting contribute to this variance.

## Discussion

In the present study, we introduce MEDIC, an AI-driven system that integrates first-generation LLMs, domain-specific expertise and human-in-the-loop feedback to enhance medication direction processing within the scope of online pharmacy operations. Decomposing the complex task of interpreting and generating standardized medication directions, MEDIC substantially optimizes the DE process in an online pharmacy. During its experimental integration into the Amazon Pharmacy workflow, MEDIC led to a 33% reduction in direction-related near-miss events—a key metric for patient safety^10,52^.

This technological advancement offers multiple benefits. First, MEDIC’s reduction in near-miss events mitigates the need for pharmacist corrections and diminishes subsequent DE rework, enhancing the pharmacy’s operational efficiency^53,54^. Second, relieving the workload associated with near-miss events for DE personnel and pharmacists positively correlates with improvements in the quality of prescription processing^55^. Finally, a decrease in near-miss events serves as a proactive approach to mitigating prescription errors, as such events are thought to be more frequent than medication errors that reach the patient but share similar root causes^56^.

The portability of MEDIC to other pharmacies or its potential availability as an external service positions it as a readily applicable solution for similar organizations. Its design, built upon mostly synthetically generated directions, along with publicly available datasets, eliminates any constraints on its applicability beyond Amazon Pharmacy. Additionally, the current fine-tuning framework, leveraging synthetic data, offers a valuable opportunity for tailoring the model to specific use cases, such as integration into a prescriber’s workflow or EHRs, among others.

In assessing the limitations of our study, several aspects warrant acknowledgment. To begin, a key limitation is the absence of direct patient feedback on the AI-enhanced medication directions. Challenges in consistent patient reporting and evaluating the effectiveness of directions highlight the need for improved error reporting and patient engagement in pharmacies^26,57^, suggesting a valuable area for future research in AI-facilitated pharmacist-patient communication. Moreover, MEDIC primarily addresses electronic prescriptions, leaving other mediums such as fax, scanned documents or oral directives relatively unexplored. These mediums, often processed manually, are more error prone, indicating the potential for integrating AI tools such as Optical Character Recognition and Speech-to-Text to manage these prescriptions within the MEDIC framework. Last, the focus of this study on single-line medication directions, which represent the majority of prescriptions, also narrows its scope. Multi-line and more-complex directions, which carry a higher safety risk, are areas ripe for future exploration to extend the applicability of our findings and develop comprehensive solutions for these more intricate scenarios.

Building on the limitations identified, MEDIC is designed to evolve through human interaction. It already incorporates semi-autonomous updates to its data-augmentation libraries and modifications to its safety guardrails and medication database (Methods). To further refine its efficacy, there is potential for enhancing MEDIC by integrating real-time feedback during DE. Techniques such as reinforcement learning from human feedback present a promising yet unexplored avenue to enrich MEDIC’s capabilities^58^.

With regard to integration of LLMs, several avenues remain unexplored for augmenting MEDIC. Initially, state-of-the-art LLMs could refine raw medication directions at the input stage, and subsequently in the third stage, assist in assembling extracted entities before safety guardrails are applied. Beyond these enhancements, an especially intriguing possibility involves the incorporation of a fine-tuned LLM as an overlay to MEDIC. This would facilitate a chatbot interface, allowing nontechnical users to query specific aspects of prescriptions or explore more general medication-related issues.

A major challenge in deploying LLMs for high-stakes tasks such as medication direction processing is the subtle control of output to prevent hallucinations or fabricated information. Balancing coherent text generation against the prevention of inaccurate outputs involves complex tradeoffs. Overly stringent controls can yield conservative, less-fluent outputs, whereas lenient measures risk introducing unreliable directions. The issue is exacerbated given the current limitations in assessing LLMs’ confidence levels using established machine-learning metrics^36^. In MEDIC, we address this challenge in part by confining the LLM’s operational scope and implementing a deterministic layer of guardrails empowered by validated pharmacy logic and gold standard data. We hope this strategy to be a case study for similar high-stakes settings and spur further research into identifying and mitigating hallucinations in LLMs^37^.

In conclusion, this study highlights the critical role of integrating machine-learning and domain expertise to address complex, high-stakes challenges in pharmacy operations. Given the magnitude of prescriptions received daily, often fraught with inaccuracies, a data-driven support system such as MEDIC becomes indispensable. It enhances operational efficiency while reducing error risks, thereby allowing pharmacists and technicians to focus on the primary goal of ensuring patient safety and well-being.

## Methods

This research complies with all ethical regulations. The research was approved by all relevant review committees at Amazon.

In this section, we outline the methods used to build and assess our AI systems, segmented into four key subsections: prescription workflow, datasets, machine-learning (ML) approaches and evaluation metrics.

### Processing and understanding prescriptions

#### Prescription processing workflow

Prescriptions can be transferred between prescribers and pharmacies through an assortment of channels, including paper, fax, phone calls and electronic prescriptions (e-scribes). Notably, e-scribes, which primarily involve the transmission of prescription or related information through an e-prescribing network, are rapidly gaining prevalence and becoming the dominant method^60^. The prescription processing workflow typically starts with two primary steps of DE and verification.

Regardless of the transmission method used, all prescriptions necessitate digitization and typing into a pharmacy’s computer system or database, a process commonly known as DE. Certified pharmacy technicians are tasked with manually transcribing the prescribers’ intentions by referencing raw prescription data. The need for this arises because prescribers frequently communicate their directives using abbreviations, terminologies and pharmacy-specific jargon. These need to be interpreted, adjusted and retyped in a standardized, succinct and patient-friendly format to facilitate efficient pharmacist approval and clear patient understanding. Given the labor-intensive and error-prone nature of current DE practices, maintaining high levels of accuracy and efficiency is crucial for optimal medication management and patient safety.

Upon the DE phase’s completion, the typed prescriptions and associated medical data undergo a meticulous review by pharmacists in a process known as pharmacist verification. This involves validating the patient’s information, prescribed medication, directions and potential interactions with any other medications the patient may be consuming. If any inaccuracies or concerns emerge during this verification process, the pharmacist sends back the prescription for correction, possibly necessitating communication with the prescribing healthcare provider for clarification or resolution before another PV step. This scenario is known as a near-miss event as a medication error is identified and corrected before reaching the patient, thereby averting potential harm. Near-misses serve as a valuable self-assessment indicator for evaluating the quality of pharmacy operations^24^. Upon successful pharmacist verification, the medication proceeds to the next downstream phase, where the emphasis is mostly on preparing, labeling, packaging and delivering to customers (see Fig. 2a for details). Like DE, pharmacist verification is largely a manual process, laden with potential for time consumption and errors. Consequently, our primary objective in this paper is to alleviate the pharmacist verification workload by enhancing the accuracy and quality of the DE phase.

Beyond the DE and pharmacist-verification stages discussed above, an additional layer of scrutiny exists in the form of a patient safety team. This team is composed of pharmacists and pharmacy quality specialists, who are certified pharmacy technicians with specialized training in medication error evaluation. The team’s core mandate is to continually assess and elevate the quality of pharmaceutical services by using a systematic approach to identifying, analyzing and mitigating medication errors. Their involvement complements the DE and pharmacist-verification phases, serving as a safeguard to ensure the attainment of optimal medication management and heightened patient safety.

#### Understanding medication directions

A medication direction is a set of instructions given by a healthcare provider that details how a patient should take or use a prescribed medication. These instructions are typically written on the prescription and are intended to be followed by the patient. In its simplest form, a medication direction comprises the five core components of verb, dose, route of administration, frequency and possibly including additional auxiliary information. For instance, the direction ‘take one tablet by mouth once daily for pain’ includes the verb (take), dose (one tablet), route of administration (by mouth), frequency (once daily) and auxiliary information (for pain); however, certain medications may not require all the information, resulting in directions like ‘apply topically to the affected area twice daily’ or ‘to be administered via the insulin pump’.

The majority of the directions, such as ‘take one tablet by mouth once daily for pain’, are deemed single-line, where each of the core components conveys a single piece of information. On the other hand, the direction ‘take one tablet by mouth in the morning and two tablets before bedtime’ comprises two separate pieces of information for the dose (one tablet and two tablets) and frequency (in the morning and before bedtime) and thus, qualifies as a multi-line direction. We note that directions such as ‘take one tablet by mouth twice daily in the morning and before bedtime’ are also considered single-line, as ‘twice daily in the morning and before bedtime’, despite its complexity, is treated as a single piece of information for the frequency component. Based on our data, over 98% of medication directions processed are single-line, which is the primary focus of this paper. Although we recognize the increased safety risks associated with multi-line directions, our current concentration on single-line formats represents an important step toward addressing the more-complex challenges posed by multi-line medication directions in future research.

### Data

In this study, we utilize two distinct, anonymized datasets for research purposes.

#### Directions set

A random subsample of processed medication directions data is extracted from a year’s worth of historical single-line directions from Amazon Pharmacy. This dataset undergoes formatting and cleaning processes to eliminate nonvalid prescriptions, such as those lacking medication directions or drug information, resulting in a representative dataset for training and quantitative evaluation of our solution against alternative benchmarks. Following these initial data cleaning processes, the final directions dataset comprises a total of approximately 1.6 million single-line samples. Each sample is characterized by the following fields: (1) ID, identifier of a unique medication direction; (2) drug ID, internal identifier of a drug available in the catalog; (3) directions, raw digital directions from prescribers; and (4) typed directions, archived prescriptions typed by pharmacy technicians and verified by pharmacists (see Supplementary Fig. 1 for more details).

We partition these data randomly into four subsets, ${{{{\mathcal{D}}}}}_{{{{\rm{H}}}}}$, ${{{{\mathcal{D}}}}}_{{{{\rm{Train}}}}}$, ${{{{\mathcal{D}}}}}_{{{{\rm{Test}}}}}$ and ${{{{\mathcal{D}}}}}_{{{{\rm{Eval}}}}}$, which are used to train and evaluate different ML models. The ${{{{\mathcal{D}}}}}_{{{{\rm{H}}}}}$ and ${{{{\mathcal{D}}}}}_{{{{\rm{Eval}}}}}$ subsets comprise 1,000 and 1,200 samples, respectively. These subsets are designated for human labeling and evaluation. The remaining data are allocated to ${{{{\mathcal{D}}}}}_{{{{\rm{Train}}}}}$ with approximately 1.58 million samples and ${{{{\mathcal{D}}}}}_{{{{\rm{Test}}}}}$ with around 20,000 samples. We ensure that each dataset maintains a representative sample of different types of directions through stratified randomization. The stratification process is guided by clustering a lower-dimensional representation of all distinct raw directions found in the directions set. In the following subsections, we detail the process of labeling the ${{{{\mathcal{D}}}}}_{{{{\rm{H}}}}}$ dataset and its further augmentation to yield three datasets: ${{{{\mathcal{D}}}}}_{{{{\rm{HL}}}}}$, ${{{{\mathcal{D}}}}}_{{{{\rm{HLA}}}}}$ and ${{{{\mathcal{D}}}}}_{{{{\rm{HLAT}}}}}$.

#### Medication catalog

In addition to assembling the direction set, we curated and integrated a comprehensive dataset containing primary medication attributes, such as strength, active ingredient and dosage form. This dataset powers a series of deterministic guardrails for algorithms, either constraining their behavior in case of errors or accurately augmenting incomplete information from incoming prescriptions.

We created the medication catalog from three main sources. The first source originates from RxNorm, a resource by the National Library of Medicine^41^, detailing information at the National Drug Code (NDC) and RxNorm Concept Unique Identifier (RxCUI) levels. As RxNorm’s weekly partial and monthly full updates may not keep pace with the rate that new NDCs are approved by the US Food and Drug Administration (FDA), we integrate new NDCs into the RxNorm from the daily-updated OpenFDA^42^ National Drug Code Directory using their corresponding RxCUI code. We automated ingestion of these two sources using the RxNorm application programming interface (API) and our custom APIs, leading to a daily-updated comprehensive dataset, covering ~99% of Amazon Pharmacy medications. We next subjected these data to a cleanup phase, discarding drug records with missing or invalid NDC values and character anomalies in numeric fields. These data were then integrated with a third source, Amazon Pharmacy’s drug catalog, which is curated by domain experts, including pharmacists and certified technicians. The final product is a comprehensive, medication-level dataset comprising (1) drug ID; (2) medication description; (3) a breakdown of the five main entities in a typical medication direction (verb, dose, route, frequency and auxiliary), indicating which are required and which are optional in a typical direction; (4) for the required entities in (3), the default or preferred information (for example, the verb ‘chew’ instead of ‘take’); and (5) primary properties of the medications, such as their active ingredients and strengths. We denote this dataset by ${{{{\mathcal{D}}}}}_{{{{\rm{MedCat}}}}}$.

### Creating MEDIC

Our primary AI-driven system, termed MEDIC, serves two critical roles. Primarily, it suggests accurate and standardized medication directions drawing on the original prescription data. In addition, it flags potential inconsistencies detected between the directions transcribed by DE technicians and those originally prescribed by the provider.

#### Suggestion

The primary objective of the ‘suggestion’ facet is to generate accurate medication directions that faithfully represent the original prescriber’s instructions, while also integrating relevant medication information. This is achieved through a three-stage process executed by MEDIC. The initial stage, pharmalexical normalization, utilizes an extensive library of pharmacy knowledge to format and standardize the raw directions through a rule-based preprocessing model. This process ensures consistency, eradicates potential errors and produces suitable input for the identification of core components in the subsequent stage. The following stage, AI-powered extraction, uses a cutting-edge transformer model, fine-tuned with unique pharmacy data, to detect and extract these core components from the incoming e-scribe direction. The concluding stage, semantic assembly and safety enforcement, synthesizes these components into meaningful, standardized and semantically accurate medication directions, using an informed blend of pharmacy knowledge, medication catalog and patient safety guardrails. The result of this three-stage process is an accurate and standardized medication direction that represents the original instruction and is designed for immediate use, necessitating little-to-no human intervention. This substantially lessens the risk of DE fatigue and potential error incidence.

#### Alternative AI approaches

Alternative AI strategies for the suggestion task include fine-tuning an LLM or using few-shot learning with an LLM. Fine-tuning involves adjusting an LLM to specialize in translation tasks, utilizing labeled data pairs of prescriber directions (inputs) and pharmacist-verification equivalents (outputs). On the other hand, few-shot learning necessitates precise LLM prompting, incorporating a small set of input–output direction examples. We designate the former method as T5-FineTuned and the latter as Claude, using both as benchmarks to assess MEDIC’s performance.

#### Flagging

The function of ‘flagging’ is to assess the equivalency of two medication directions, achieved through a component-wise comparison of all core components. The model’s operational flow can be partitioned into multiple phases. Initially, both directions undergo the same two stages of pharmalexical normalization and AI-powered extraction, described above. Following this, the extracted components are standardized within a post-processing layer utilizing the medication catalog. The semantic information pertaining to each core component is then contrasted across both directions. Should any component exhibit differing semantic information, it is flagged. The ultimate output of the flagging process is a Boolean variable that signals whether the two medication directions are equivalent or not. On the whole, flagging serves as a proactive measure against direction errors, effectively identifying and underscoring potential inconsistencies between the directions typed by the DE technicians and the original medication directions.

Subsequently, we delve into the architecture of MEDIC. Given that our approach to flagging is a straightforward application of the modules developed in solving the suggestion task, our exposition will primarily focus on the three-part process that constitutes the suggestion task: pharmalexical normalization, AI-powered extraction and semantic assembly and safety enforcement.

#### Pharmalexical normalization

The raw data for medication directions often contain noise that makes it challenging to use in its original form. These directions can include a mix of everyday English, pharmacy-specific jargon, abbreviations and even typographical and grammatical errors. To make these data usable by NLP models, it is crucial to convert the original text into a clean, simplified English version of the directions.

This process goes beyond standard preprocessing steps such as lowercasing, stop-word removal, deduplication and spell correction. We developed a customized text normalization strategy specifically designed for medication directions, informed by our review of samples from historical prescriptions and the expert knowledge of pharmacists.

This unique text normalization process applies a sequence of atomic, pattern-based transformations, each guided by one of hundreds of pharmacist-verification rules. The result is a set of high-quality, consistent and accurate medication directions that are readily comprehensible by both humans and downstream ML models. Examples of inputs and outputs of this process are shown in Fig. 2b,c and Supplementary Table 1. We conduct a sensitivity analysis to assess the impact of this individual processing step, both in isolation and in conjunction with subsequent stages.

#### AI-powered extraction

The central pillar of MEDIC is an adapted named entity recognition (NER) model, precisely tailored for the unique demands of pharmacy directions. This model is a fine-tuned, faster version of the renowned BERT transformer architecture (DistilBERT)^43,44^, used to identify and extract core components within a medication direction. The guiding principle of this fine-tuning process is to construct an NER model capable of faithfully extracting information from the incoming direction exactly as the prescriber wrote, regardless of any errors present in the original direction. Any potential corrections are left to the subsequent stage, semantic assembly and safety enforcement.

The primary obstacle in executing this fine-tuning lies in the construction of appropriate training data. Specifically, a set of medication directions is needed where each core component is accurately labeled. We utilize two sources to produce and refine these data. Initially, we manually label the set ${{{{\mathcal{D}}}}}_{{{{\rm{H}}}}}$ of approximately 1,000 historical directions with the assistance of pharmacy quality specialists and denote the labeled set by ${{{{\mathcal{D}}}}}_{{{{\rm{HL}}}}}$. Extended Data Table 6 provides examples of these data labels. Second, we capitalize on ${{{{\mathcal{D}}}}}_{{{{\rm{HL}}}}}$ to generate a considerably larger augmented dataset ${{{{\mathcal{D}}}}}_{{{{\rm{HLA}}}}}$, comprising 10,000 synthetically created labeled directions. We also conduct sensitivity tests to emphasize the value and optimal size of ${{{{\mathcal{D}}}}}_{{{{\rm{HLA}}}}}$, balancing both accuracy of the model and training time.

##### Data labeling

The construction of ${{{{\mathcal{D}}}}}_{{{{\rm{HL}}}}}$ would need to balance multiple objectives. On one hand, the labeled entities should be well defined and interpretable to make the human labeling phase manageable. On the other hand, they should account for natural variations among different prescriptions. From the core components, the most complex one is the ‘auxiliary information’, given the broad set of instructions and words that prescribers can include in it. Based on the most common instructions seen in the data, we further break the auxiliary information into five sub-entities. In summary, the array of potential entities requiring labeling in each direction is presented in Extended Data Table 7, while their hierarchical organization is illustrated in Extended Data Fig. 1.

Leveraging the labeled data, we create several libraries for use in the next (data augmentation) phase. For each of the nine components previously discussed, we generate a library of its unique possible values as they appear in ${{{{\mathcal{D}}}}}_{{{{\rm{HL}}}}}$. These are denoted as ${{{{\mathcal{L}}}}}_{{{{\rm{verb}}}}},{{{{\mathcal{L}}}}}_{{{{\rm{dose}}}}},\ldots ,{{{{\mathcal{L}}}}}_{{{{\rm{freq}}}}},{{{{\mathcal{L}}}}}_{{{{\rm{aux}}}}{{\mbox{-}}}{{{\rm{indic}}}}},\ldots ,{{{{\mathcal{L}}}}}_{{{{\rm{aux}}}}{{\mbox{-}}}{{{\rm{period}}}}}$. These nine libraries are subsequently expanded by incorporating potential values identified by pharmacy quality specialists. Ultimately, for every direction in ${{{{\mathcal{D}}}}}_{{{{\rm{HL}}}}}$, we form a unique pattern, exemplified in the rightmost column of Extended Data Table 6. The collection of all unique patterns acquired in this manner forms the pattern library, referred to as ${{{{\mathcal{P}}}}}_{{{{\rm{HL}}}}}$.

##### Data augmentation

Considering the high cost of manual labeling and the potential need for a larger labeled training dataset for BERT fine-tuning, we resorted to data augmentation. This approach enabled us to create artificially generated prescription samples that not only enhance the robustness and confidence of our extractions but also substantially broaden the scope of our training set.

In ${{{{\mathcal{D}}}}}_{{{{\rm{HLA}}}}}$, we generated a new sample by first randomly selecting a pattern *p* from ${{{{\mathcal{P}}}}}_{{{{\rm{HL}}}}}$. For each component *c* in *p*, we randomly choose an element from the respective library ${{{{\mathcal{L}}}}}_{c}$. We then combine these selected components in the same order as they appear in *p* to generate a synthetic direction which is added to ${{{{\mathcal{D}}}}}_{{{{\rm{HLA}}}}}$. This process, repeated 10,000 times, allows us to expand ${{{{\mathcal{D}}}}}_{{{{\rm{HL}}}}}$ tenfold, yielding 10,000 samples in ${{{{\mathcal{D}}}}}_{{{{\rm{HLA}}}}}$, each one composed of a varying number of components as well as number of tokens, including words, numbers, punctuation and symbols (Supplementary Fig. 2a,b and Supplementary Table 2).

It is important to note, however, that not all synthetically generated directions in ${{{{\mathcal{D}}}}}_{{{{\rm{HLA}}}}}$ might be realistic or clinically consistent. But, bearing in mind our main guiding principle, the primary objective of extraction is to accurately capture what the prescriber has written. Furthermore, we have observed instances of incorrect prescriptions from prescribers. To handle such cases, we focused on accurately identifying all potential erroneous entities. We then contrasted these entities with the medication catalog available in the third phase of semantic assembly and safety enforcement, granting us full control over these incorrect cases.

##### Extraction testing set

To evaluate the performance of the NER operations performed by MEDIC, we used ${{{{\mathcal{D}}}}}_{{{{\rm{HLAT}}}}}$, a testing dataset with 10,000 samples generated following the same methodology as in ${{{{\mathcal{D}}}}}_{{{{\rm{HLA}}}}}$ (see Supplementary Fig. 2a–d,f and Supplementary Table 2 for details on ${{{{\mathcal{D}}}}}_{{{{\rm{HLA}}}}}$ and ${{{{\mathcal{D}}}}}_{{{{\rm{HLAT}}}}}$.)

##### Evaluation

The confusion matrix (Supplementary Fig. 3e) underscores the accuracy of MEDIC’s AI-powered extraction. Out of a total of 160,484 entities available across the 10,000 directions generated in the synthetically augmented test dataset ${{{{\mathcal{D}}}}}_{{{{\rm{HLAT}}}}}$, only six were misclassified: one from the dose component, four from frequency and one from the route component. The average precision, recall and F1 score all exceeded 0.99.

Furthermore, our sensitivity analysis underscores the critical role of data augmentation in model performance. Training the NER model exclusively with the 1,000 samples in ${{{{\mathcal{D}}}}}_{{{{\rm{HL}}}}}$ resulted in a marked decline in the F1 score to approximately 0.70. In optimizing the number of augmented samples, we found that an F1 score of 0.90 was attained with a reduced set of 5,000 augmented samples. Notably, substantially larger augmentation sets (for example, 50,000 and 100,000) do not yield statistically significant performance variations across all evaluated metrics when compared to an augmentation size of 10,000 for ${{{{\mathcal{D}}}}}_{{{{\rm{HLA}}}}}$.

##### Hyper-parameter optimization

For the fine-tuning of BERT, we optimized four primary hyper-parameters and set the batch size to 16, set the learning rate to 1 × 10^−4^, configured the model to undergo three training epochs and applied a weight decay factor of 1 × 10^−5^. These choices were the result of testing three main techniques: Bayesian optimization^61^, derivative-free-optimization^62^ and a simple grid search. All three approaches yielded similar parameters; however, we opted for Bayesian optimization for the MEDIC production implementation due to its efficient convergence.

#### Semantic assembly and safety enforcement

We used post-processing on the outcomes of AI-powered extraction, leveraging the medication catalog and pharmacy expertise. This crucial step ensured patient safety by preventing suggestions that might be inaccurate or even harmful.

##### Suggestion assembly

Initially, the medication catalog is used to fill in any missing essential components in the direction. These data specify which core components are required for a given drug ID (the list of all possible core components is in Extended Data Table 7). If any of these core components are missing from the output of AI-powered extraction, either due to omission in the original direction or an extraction failure by the NER model, and if a value for the missing component is available in the medication catalog, that value is added to the list of extracted entities from AI-powered extraction. Following this, all the extracted entities are compiled to create a preliminary direction. They are ordered as follows: ‘verb’, ‘dose’, ‘route’, ‘frequency’, ‘auxi-indic’, ‘auxi-time’, ‘auxi-period’, ‘auxi-action’ and ‘auxi-max dose’. An example is illustrated in Fig. 2b.

##### Patient safety guardrails

To ensure the utmost patient safety, we have implemented a set of guardrails within the MEDIC pipeline. These safety measures are designed to prevent the generation of potentially harmful suggestions. Incorporated as independent layers, they allow for easy integration, modification or decommissioning. These guardrails, developed in collaboration with pharmacists and quality specialists, are tailored to enhance the operation of MEDIC. They are listed below in their order of implementation. Should any of these guardrails be triggered, MEDIC will immediately cease operation, opting not to generate a suggestion.

- Any discrepancy between the values of the nine components extracted by AI-powered extraction and those available in medication catalog halts the generation of a suggestion. This guardrail operates on a fundamental principle: medication catalog, depending on the specific drug ID, may only have values for a limited subset of the nine components; however, these values serve as a crucial verification mechanism for the output of AI-powered extraction. If a discrepancy arises between the output generated by AI-powered extraction and the medication catalog, this incongruity is considered symptomatic of a potential error in the AI-powered extraction algorithm. For instance, should AI-powered extraction identify the verb ‘apply’ while the medication catalog specifies ‘take’—especially in cases where the dosage form is a ‘tablet’—this triggers an alert. As a risk-mitigation strategy, we elect to suspend reliance on any extractions produced by AI-powered extraction and consequently halt MEDIC from proposing a direction.
- The suggestion generation is aborted if AI-powered extraction extracts multiple values for any of the nine components. This precautionary measure is put in place because MEDIC is specifically trained and optimally performs on single-line prescriptions and may extract multiple values in multi-line directions.
- A suggestion may not be generated if there is a value for the ‘dose’ but no corresponding value for the ‘verb’. This decision depends on the drug-specific requirements outlined in the medication catalog.
- In cases where no value for the ‘frequency’ component is extracted, the suggestion generation is halted. This guardrail helps prevent the creation of potentially incorrect suggestions that are missing key information, which cannot be inferred from the raw direction.
- When there is no value for the ‘dose’ component and the dosage form is either ‘tablet’ or ‘capsule’, suggestion generation is halted. This guardrail aids in preventing the creation of potentially incorrect suggestions due to missing crucial information.

A detailed schematic depicting the three main stages of MEDIC, the datasets used and the interaction with pharmacists and domain experts is given in Supplementary Fig. 3.

### Evaluations

We assess the efficacy of MEDIC using both retrospective (or offline) and prospective (or online) evaluation methodologies. In the retrospective evaluation, we benchmark MEDIC against T5-FineTuned, Claude and a rule-based model. This assessment leverages recognized ML and NLP metrics and is complemented by patient safety evaluations performed by humans. Additionally, this retrospective evaluation probes the strengths and limitations of cutting-edge LLMs when crafting medication suggestions. For the prospective evaluation, we compare MEDIC with a previously established algorithm operational within Amazon Pharmacy, through a before-and-after study. This comparison gauges MEDIC’s impact on pharmacy quality metrics. The decision to conduct this in-production experimental assessment of MEDIC was made solely after the system met the required safety criteria during the retrospective evaluations.

#### Retrospective comparisons

We started by measuring the performance of MEDIC to detect and extract the nine potential components in each direction within ${{{{\mathcal{D}}}}}_{{{{\rm{HLAT}}}}}$ using traditional ML metrics of precision, recall, F-score and an analysis of the confusion matrix, providing classification statistics for all components.

##### NLP evaluations

Following this, we benchmarked the performance of MEDIC suggestions against three other methods: rule-based, four variations of T5-FineTuned and the most promising variant of Claude. We selected the most advanced version of Claude for this evaluation, considering its high latency and costs when applied to a large dataset of 20,000 cases, as in ${{{{\mathcal{D}}}}}_{{{{\rm{Test}}}}}$. Detailed descriptions of these benchmarks will be provided in subsequent sections. Initially, for every incoming direction within the ${{{{\mathcal{D}}}}}_{{{{\rm{Test}}}}}$ set, we utilized each of the methods (MEDIC, rule-based, all variations of T5-FineTuned and a ten-shot version of Claude) to generate a respective suggestion. Following this, we gauged the quality of the generated suggestions from each model using two widely accepted NLP machine-translation metrics, BLEU^63^ and METEOR^64^.

##### Description of BLEU and METEOR

BLEU and METEOR are evaluation metrics in NLP that quantitatively measure the similarity between machine-generated text and human-provided reference text^63,64^. BLEU scores range from 0 (no overlap with the reference) to 1 (perfect match), whereas METEOR scores also range between 0 and 1 but account for synonymous matches, stemming and word order, providing a more holistic comparison^64^. Typically, a BLEU score above 0.7 is considered close to human-level performance for certain tasks^65^, whereas METEOR scores nearing 0.9 indicate high-quality translations. Nevertheless, machine translations often trail human translations, which can achieve near-perfect scores on both metrics^65^.

##### Metrics limitations

Examples highlighting limitations of BLEU and METEOR metrics in the context of patient safety can be seen in Supplementary Table 3. In compliance with Amazon confidentiality policies, the examples of ground-truth directions provided here are synthesized but crafted to closely mirror the style and information of the original directions.

##### Evaluation results for BLEU and METEOR

Supplementary Fig. 4a corroborates the importance of fine-tuning data size. Specifically, T5-FineTuned benchmarks based on the T5 architecture, namely T5-FineTuned (100), T5-FineTuned (1K), T5-FineTuned (10K), T5-FineTuned (100K) and T5-FineTuned (1.5M), display a positive correlation between the size of the training set and performance metrics. Optimal performance, characterized by BLEU = 0.74 and METEOR = 0.87, is achieved with a training set size of 1.5M samples from ${{{{\mathcal{D}}}}}_{{{{\rm{Train}}}}}$; however, challenging the growing belief that fine-tuning foundational models on small amount of labeled data suffices for optimal outputs^44–46^, the performance of the T5-FineTuned (100) model actually lags behind even that of the rule-based output of the pharmalexical normalization stage. This underscores the indispensability of ample training data in our pharmacy context.

Moreover, while MEDIC and benchmark T5-FineTuned (1.5M) present analogous performance metrics, the latter possesses a slight edge. This advantage stems from its capability at grammar correction (for instance, rectifying typos such as ‘two tablet’) and its competence in navigating intricate auxiliary information. Given MEDIC’s built-in safety constraints, which inhibit suggestions for around 20% of the cases, termed the ‘MEDIC Inactive’ set, we tailored our focus in Supplementary Fig. 4b to the 80% of prescriptions for which MEDIC did provide suggestions, called the ‘MEDIC Active’ set, and the findings echo our previous observations (for confidentiality reasons, the ratios of MEDIC Active and MEDIC Inactive set are rounded to the nearest multiple of 20%).

Notably, Supplementary Fig. 4b also illuminates the data efficiency of the AI-powered extraction stage of MEDIC, which is designed to identify entities rather than constructing entire directions, unlike T5-FineTuned (1.5M). Specifically, MEDIC was provided with a meager set of 1,000 labeled samples (${{{{\mathcal{D}}}}}_{{{{\rm{HL}}}}}$), which was then synthetically augmented to ${{{{\mathcal{D}}}}}_{{{{\rm{HLA}}}}}$, as opposed to T5-FineTuned (1.5M), which required 1.5M labeled samples in ${{{{\mathcal{D}}}}}_{{{{\rm{Train}}}}}$. While MEDIC further leverages ${{{{\mathcal{D}}}}}_{{{{\rm{MedCat}}}}}$ in ‘Semantic assembly and safety enforcement’, the benefit from ${{{{\mathcal{D}}}}}_{{{{\rm{MedCat}}}}}$ is manifested outside the MEDIC Active set.

Additionally, our sensitivity analysis showed that the performance remained the same, irrespective of whether T5-FineTuned models were inputted with outputs from the rule-based pharmalexical normalization.

##### Human review

In a similar vein, we applied MEDIC and the best variations of T5-FineTuned and all versions of Claude on the ${{{{\mathcal{D}}}}}_{{{{\rm{Eval}}}}}$ set and submitted the outputs from all models for a human evaluation. The primary evaluation criterion here is the rate of suggestions with ‘critical errors’, defined as suggestions that would lead to a near-miss event in a real-world production environment if used by technicians. Pharmacists further assessed the near-misses identified in this human review to gauge their clinical severity and potential patient harm, taking into account the specific medications involved in each case.

#### Rule-based benchmark

This benchmark formulates a suggestion from the incoming direction after it has been processed by the pharmalexical normalization module within the MEDIC framework. The inclusion of this benchmark also functions as a sensitivity analysis for the initial stage of MEDIC, examining its potential as a standalone model.

#### T5-FineTuned benchmarks

For these comparative benchmarks, we enlisted the functionality of the text-to-text transformer (T5). This transformer-based architecture, which is specifically engineered for text-to-text tasks such as language translation^66^, is a natural candidate to address the task of generating high-quality suggestions (the output or translated text) from nonstandard or low-quality incoming directions (the input or raw text). This approach, influenced by recent research in pharmacy^67,68^, serves as an important benchmark for MEDIC. In addition, and in line with the growing body of literature demonstrating the ability of LLMs to be fine-tuned for a variety of tasks using small data, we implement this technique with varying fine-tuning data sizes. More specifically, we use the (base) version of T5 transformer^69^ and fine-tune it with *n* stratified random (input–output) pairs from ${{{{\mathcal{D}}}}}_{{{{\rm{Train}}}}}$, where *n* belongs to the set {100, 1,000, 10,000, 100,000, 1,500,000}. These five models are denoted as T5-FineTuned (100), T5-FineTuned (1k), T5-FineTuned (10k), T5-FineTuned (100k) and T5-FineTuned (1.5M), respectively.

Additionally, to conduct a sensitivity analysis, we further preprocessed the inputs through the pharmalexical normalization module of MEDIC before passing them to T5-FineTuned (100), T5-FineTuned (1k), T5-FineTuned (10k), T5-FineTuned (100k) and T5-FineTuned (1.5M). From the experiments, we did not observe any statistically significant differences in performance with respect to the original raw-to-ideal directions results.

#### Modern LLM benchmarks

As the deployment of LLMs and text generation systems becomes increasingly prevalent in various domains, including healthcare, concerns about the quality and reliability of the generated content have gained substantial attention^70–72^. In the specific context of medication directions, where accuracy and clarity are paramount as discussed in the previous sections, the potential for these models to produce hallucinatory or misleading information poses a critical challenge. While models such as BERT and T5, featuring hundreds of millions of parameters, are classified as LLMs^70^ and meet the runtime requirements of our high-throughput pharmacy application, their newer counterparts (boasting tens to hundreds of billions of parameters, albeit with slower runtime and elevated inference costs) demonstrate notable performance gains in a variety of tasks. Therefore, we also conducted quantitative and qualitative assessments of the strengths and weaknesses of medication direction suggestions generated by modern LLMs such as Anthropic Claude v.2.1 (ref. ^40^), ChatGPT4 (ref. ^73^) and Bard/Gemini Pro^48,74^. For both assessments, we asked the models to generate the most accurate, standard and correct medication direction given the incoming prescription, analyzing their generated outputs from the perspective of patient comprehension and safety. Starting with simple guidelines, we further refined the prompt provided to the models iteratively, obtaining the best results across all of them with the following template:


"""Suppose you are a pharmacist and you receive the following medication direction from a prescriber {insert direction here}.You need to suggest the most standard and accurate medication direction. What would you suggest in this case with no more information available.Only print the suggested direction between double quotes. Do not print additional text in the response."""


Examples for Claude few-shot learning approaches are selected via stratified sampling to provide a representative set of raw-to-ideal directions pairs to the model. As part of the qualitative and sensitive analysis, we enriched the original prompt by including the information of the medication as context, that is, including the information consolidated in ${{{{\mathcal{D}}}}}_{{{{\rm{MedCat}}}}}$ as part of the prompt, medication description, required components, preferred information (for example, verb) and properties such as strength and active ingredient(s). For this, we use the following modified prompt template:


"""Suppose you are a pharmacist and you receive the following medication direction from a prescriber {insert direction here}.You need to suggest the most standard and accurate medication direction using the following information available for the medication: {Insert medication information here}What would you suggest in this case with no more information available.Only print the suggested direction between double quotes. Do not print additional text in the response."""


#### Retrospective evaluation of the flagging model

Recall that the flagging module of MEDIC is integrated into the prescription workflow and activates in real-time when DE technicians input, update or modify the medication directions, providing an immediate alert if potential errors are detected. Suppose the incoming direction reads, ‘take one tablet by mouth daily’. The flagging model issues a warning of an incorrect dosage when the DE technician inputs ‘take two tablets’, rather than waiting for the completion of the entire direction. This enables early detection and correction of errors; however, given the dynamic and interactive nature of this process, it poses a challenge to track all real-time modifications and thus accurately measure flagging performance online. To address this, we conduct a retrospective analysis on 795 historical directions near-miss events, where direction errors are already manually labeled and corrections are clearly recorded. We apply the flagging model to these cases to assess its capability in detecting direction errors.

#### Prospective comparisons

To comprehensively test the end-to-end performance of MEDIC, wherein both the suggestion and flagging components function in synergy, we proceeded to prospective comparisons within the Amazon Pharmacy production environment, upon affirming the system’s adherence to patient safety standards in the retrospective evaluations. To this end, in collaboration with the Amazon Pharmacy Engineering and Operations teams, we have implemented MEDIC as a Health Insurance Portability and Accountability Act-compliant API. All of MEDIC’s components were built using Python and hosted utilizing Amazon Web Services cloud technology. This includes custom data ingestion and preprocessing, training and fine-tuning. These are compatible with the Huggingface package, a leading-edge toolkit for transformers^75^. Then, during an experimental phase, we substituted MEDIC for Amazon Pharmacy’s then-active production system (a hybrid of supervised learning and rule-based modules) and conducted a before-and-after comparative analysis of their respective performances (due to complexities associated with accurately replicating the then-active production system, it was excluded from our retrospective comparisons).

Our primary comparison metric was the rate of directions near-miss events^23^, defined as the proportion of directions deemed erroneous by the pharmacist-verification process and subsequently sent back to DE technicians for rectification. Additionally, we also considered secondary metrics such as (1) suggestion coverage, which represents the proportion of prescriptions for which a suggestion is generated; (2) adoption rate, the proportion of generated suggestions selected by DE technicians; and (3) edit ratio, which denotes the proportion of selected suggestions that were edited by DE technicians before finalization.

#### Continuous human-in-the-loop enhancements

A key facet of MEDIC is its provision for continuous feedback and enhancement through human evaluations. During the experimental testing of MEDIC, we instituted semi-automated mechanisms to facilitate this iterative improvement. Specifically, if a DE technician either declined a suggestion or adopted it but made substantial edits, the relevant prescription (along with the outputs from all of MEDIC’s intermediate stages) was added to a human review queue (HRQ). Additionally, prescriptions were sent to the HRQ if a suggestion, once adopted by DE technicians, subsequently led to a directions near-miss event. The HRQ was periodically reviewed with the assistance of quality specialists to diagnose the source of any discrepancies. Based on their findings, updates were made to the ${{{{\mathcal{D}}}}}_{{{{\rm{MedCat}}}}}$ or to datasets such as ${{{{\mathcal{D}}}}}_{{{{\rm{HL}}}}}$ and ${{{{\mathcal{P}}}}}_{{{{\rm{HL}}}}}$, and the various ${{{\mathcal{L}}}}$ libraries, and these updates were subsequently used to retrain AI-powered extraction.

### Reporting summary

Further information on research design is available in the Nature Portfolio Reporting Summary linked to this article.

## Online content

Any methods, additional references, Nature Portfolio reporting summaries, source data, extended data, supplementary information, acknowledgements, peer review information; details of author contributions and competing interests; and statements of data and code availability are available at 10.1038/s41591-024-02933-8.

### Supplementary information

Supplementary Information Supplementary Figs. 1–4, Tables 1–3 and Results 1–4.

Reporting Summary

## Figures and Tables

**Extended Data Table 1:**
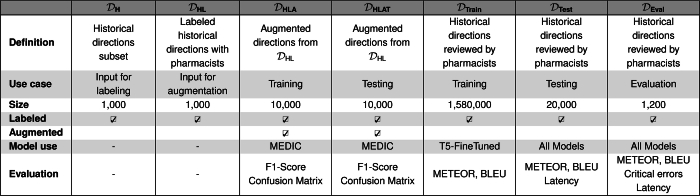
Datasets summary

Summary description of the different datasets used in the study for training and testing the AI approaches including their number of samples, techniques used to construct them, their specific use-case with the different models, and the type of metrics used to evaluate the performance of the models when performing inference with them.

**Extended Data Table 2:**
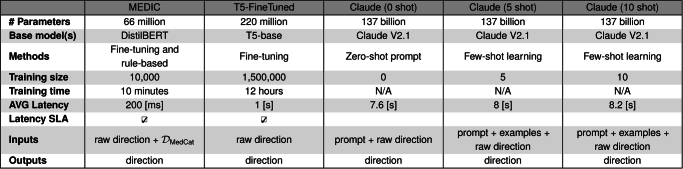
Models summary

Side-by-side comparison of the different models and the specific techniques used for training/fine-tuning them with the corresponding datasets. The latency service level agreement (SLA) row indicates if the model satisfies the production SLA imposed by the online pharmacy setting. GPU machines are used to execute Claude, while basic CPU machines are used for MEDIC and T5-FineTuned.

**Extended Data Table 3:**
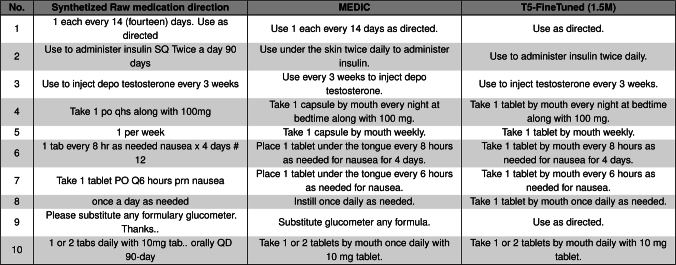
Examples of T5-FineTuned (1.5M) mistakes

Side-by-side comparison of outputs generated by MEDIC and T5-FineTuned (1.5M) from raw incoming medication directions where T5-FineTuned (1.5M) introduces wrong components in the outputs, leading to poor or incorrect medications directions. In contrast, MEDIC generates consistent and safe suggestions, validated by pharmacists.

**Extended Data Table 4:**
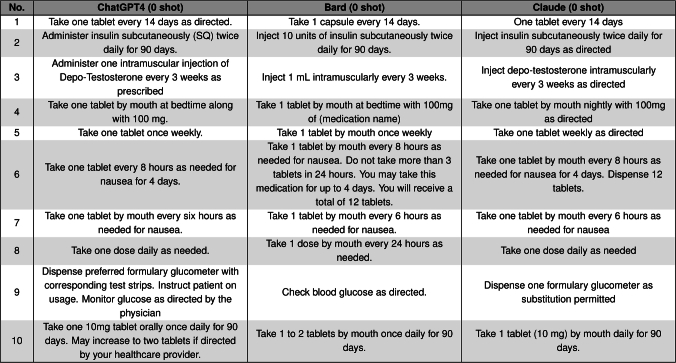
State-of-the-art chatbot models hallucination examples

Side-by-side comparison of outputs generated by ChatGPT4, Bard, and Claude models from the examples of synthetized raw incoming medication directions in Table 3.

**Extended Data Table 5:**
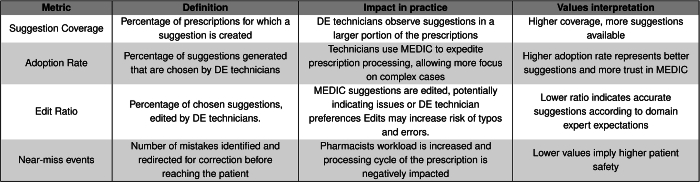
Online evaluation metrics summary and impact

Metrics used for experimental evaluation of MEDIC in Amazon Pharmacy production environment, including their practical impact and interpretation.

**Extended Data Table 6:**
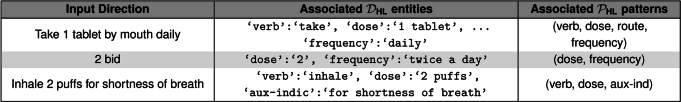
Human annotated directions examples

Examples of directions and their associated *D*_*HL*_ and *P*_*HL*_ annotations.

**Extended Data Table 7:**
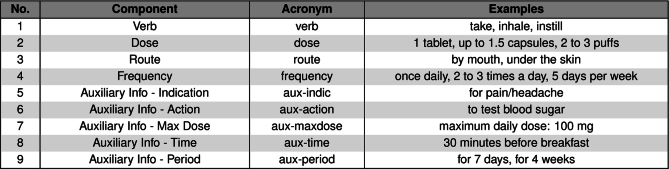
Directions components

List of potential components in each direction that is labeled in *D*_*HL*_.

## Data Availability

The RxNorm and US FDA datasets are publicly available from the National Library of Medicine website and US openFDA website, respectively (https://www.nlm.nih.gov/research/umls/rxnorm/index.html and https://open.fda.gov/). The remaining datasets generated during the study cannot be made publicly available due to Health Insurance Portability and Accountability Act and Amazon policies. Requests for access to the proprietary Amazon data used in this study will be reviewed by the corresponding author and appropriate Amazon committees to ensure compliance with intellectual property and confidentiality obligations. Interested parties may submit their requests to the corresponding author. The response time will be within approximately 30 business days. Please note that the release of individual-level prescription data may be restricted to protect patient confidentiality. Any other data and materials that can be shared will undergo a de-identification process and will be released subject to the terms of a data-use agreement.

## Code availability

All analysis was performed using open source and publicly available Python packages: pandas v.1.4.4, numpy v.1.26.0, matplotlib v.3.5.2, seaborn v.0.11.2, Sagemaker v.2.100.0, Huggingface Transformers v.4.18.0, Huggingface-hub v.0.8.1; Torch v.1.13.1+cu117, Re v.2.2.1; boto3 v.1.24.34; botocore v.1.27.59; compress-pickle v.2.1.0; nltk v.3.8; and wordcloud v.1.8.2.2. All codes were executed in Python (v.3.10.6); however, code supporting the findings of this study is not publicly available due to copyright restrictions and is the property of Amazon Pharmacy.
