# Supplementary material for: Large language models for preventing medication direction errors in online pharmacies
Source: Nat Med. 2024 Apr 25;30(6):1574–82. doi: 10.1038/s41591-024-02933-8 (PMC11186789; doi:10.1038/s41591-024-02933-8)
Supplement: Supplementary file 1 — Supplementary Figs. 1–4, Tables 1–3 and Results 1–4. [file 41591_2024_2933_MOESM1_ESM.pdf]

nature medicine

Article https://doi.org/10.1038/s41591-024-02933-8

Large language models for preventing medication direction errors in online pharmacies

In the format provided by the authors and unedited

Supplementary Information: Large language models for preventing medication direction errors in online pharmacies

Cristobal Pais, PhD[1,*], Jianfeng Liu, PhD[1], Robert Voigt, PharmD[1], Vin Gupta, MD MPA[1,2], Elizabeth Wade, PharmD[1], and Mohsen Bayati, PhD[1,3]

[1]Amazon LLC, 410 Terry Ave N, Seattle 98109, WA; [2]University of Washington, Department of Health Metrics Sciences, 1410 NE Campus Parkway Seattle, WA 98195; [3]Stanford University, Operations, Information, and Technology at Graduate School of Business, 655 Knight Way, Stanford, CA 94305

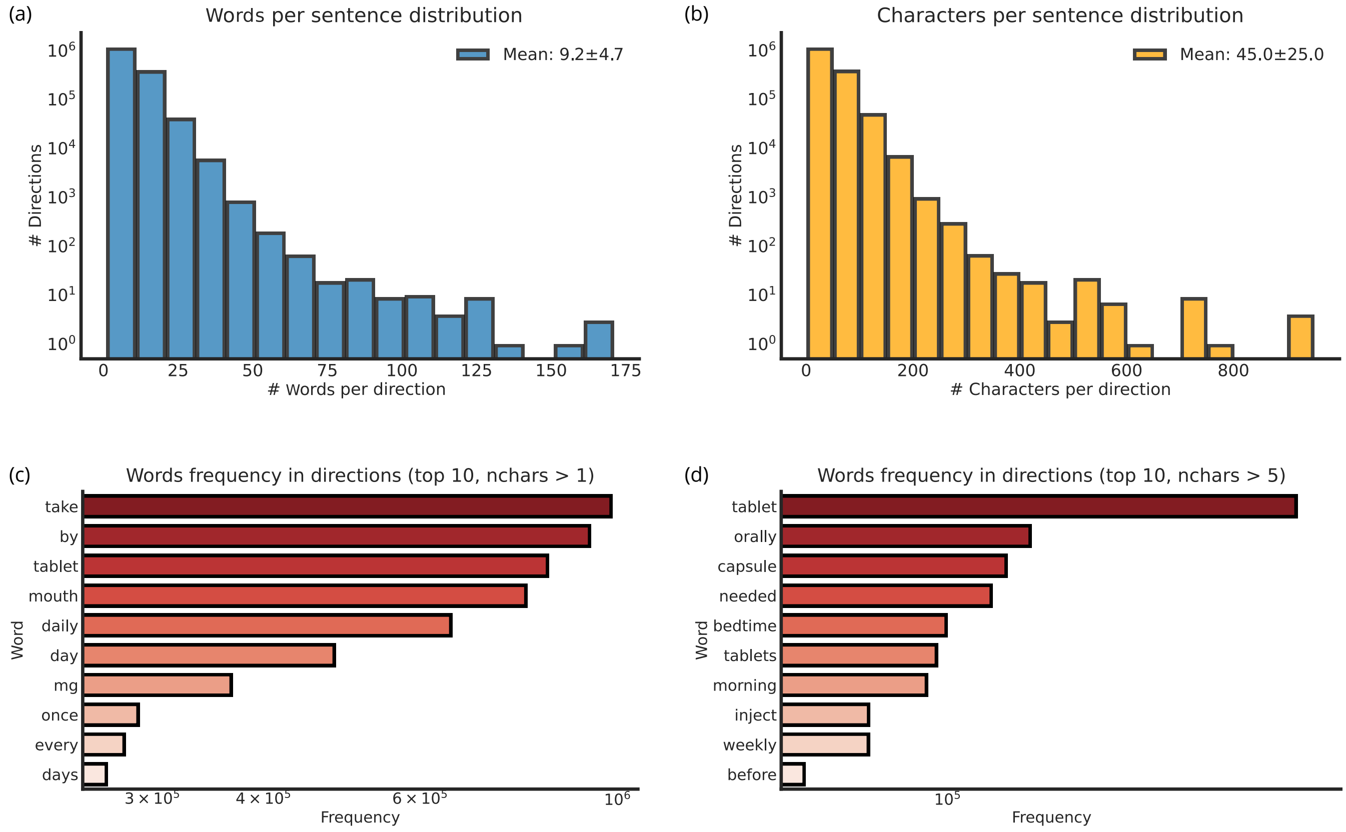

Supplementary Fig. 1. Directions set statistics. Comprised by 1.6M medication directions, the Directions set used to train and evaluate MEDIC covers directions from more than 5,000 drugs. (a) Distribution of the number of tokens/words per direction. (b) Distribution of the number of characters composing each direction inside the dataset. (c) Top ten words composed by two or more characters in the data, ordered by decreasing frequency of appearance. (c) Top ten words composed by five or more characters in the data, ordered by decreasing frequency of appearance.

Raw Direction	Normalized Direction	Changes
TK 1 TABLE PO QD.	take 1 tablet by mouth daily	`‘tk’ -> ‘take’, ‘table’ -> ‘tablet’, ‘po’ -> ‘by mouth’, ‘qd’ -> ‘daily’`
2-3 cap in am and hs	2 to 3 capsules in the morning and at bedtime	`‘2-3’ -> ‘2 to 3’, ‘cap’ -> ‘capsules’, ‘in am’ -> ‘in the morning’, ‘hs’ -> ‘at bedtime’`
Inject .5ml sc; PRN	inject 0.5 ml under the skin as needed	`‘.5ml’ -> ‘0.5 ml’, ‘sc’ -> ‘under the skin’, ‘prn’ -> ‘as needed’`

Supplementary Table 1. Examples of Pharmalexical Normalization. The Raw Direction column represents the kind of inputs provided by prescribers to the online pharmacy, including abbreviations, typos, non-standard puntuation and spacing, among other characteristics. In the middle column, we observe the outputs generated by Pharmalexical Normalization using the Raw Direction data as inputs, highlighting the specific changes in the final column.

	$\mathcal{D}_{\mathbf{HLA}}$	$\mathcal{D}_{\mathbf{HLAT}}$
Total directions	10,000	10,000
Unique tokens	1,022	1,031
Unique words	891	896
Total components	160,126	160,484
Unique components	9	9

Supplementary Table 2. AI-Powered extraction datasets. Training $\mathcal{D}_{\mathbf{HLA}}$ and testing $\mathcal{D}_{\mathbf{HLAT}}$ datasets global summary. Tokens are defined as text pieces including words, numbers, punctuation, and symbols.

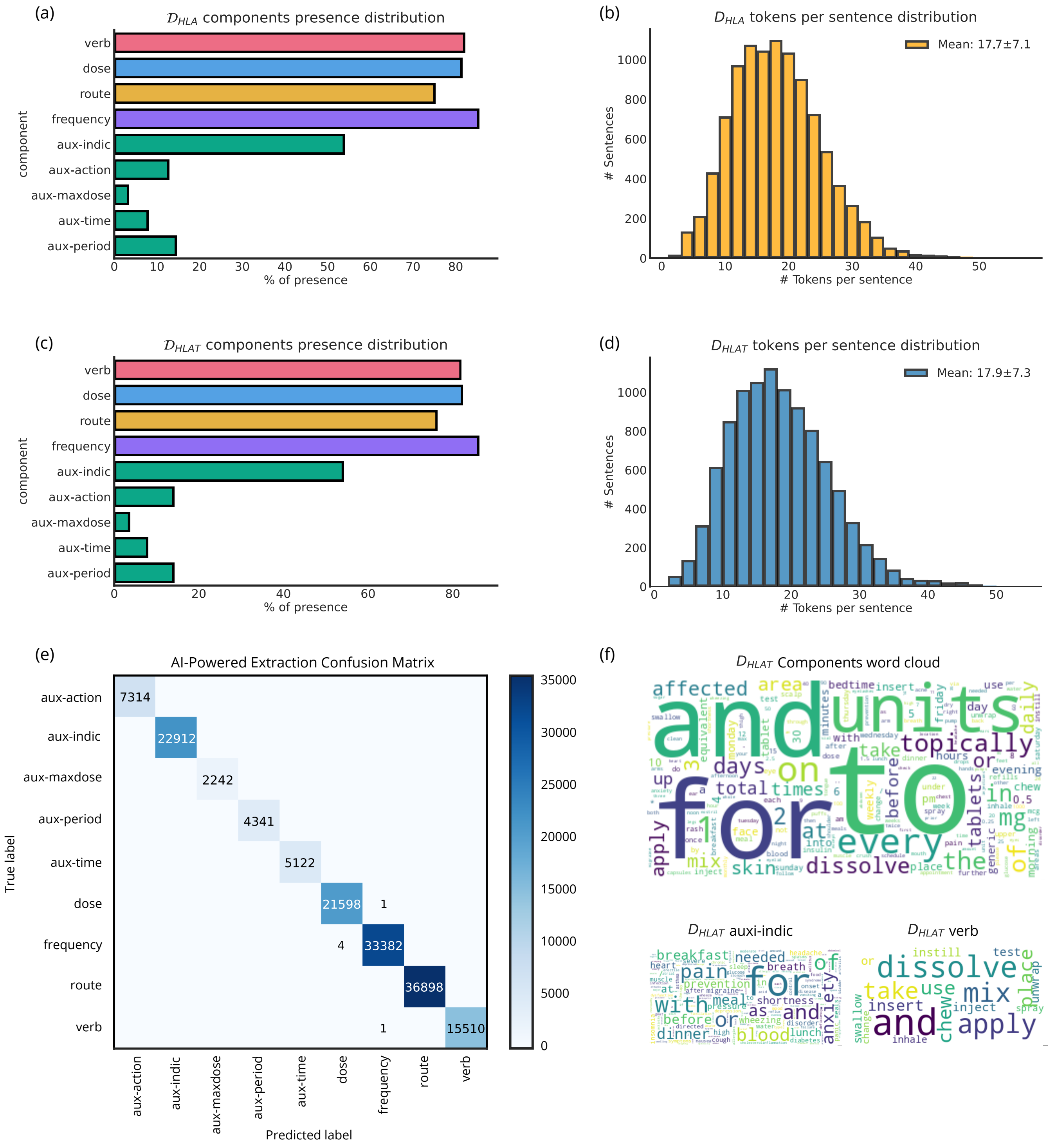

Supplementary Fig. 2. MEDIC AI-Powered extraction data summary and results on the synthetically augmented test data set $\mathcal{D}_{\mathrm{HLAT}}$. (a) Training AI-Powered Extraction data components presence across all directions in $\mathcal{D}_{\mathrm{HLA}}$. (b) Distribution of the number of tokens – including words, numbers, punctuation, and symbols – per sentence in training AI-Powered Extraction data $\mathcal{D}_{\mathrm{HLA}}$. (c) Test AI-Powered Extraction data components presence across all directions in $\mathcal{D}_{\mathrm{HLAT}}$. (d) Distribution of the number of tokens – including words, numbers, punctuation, and symbols – per sentence in testing NER data $\mathcal{D}_{\mathrm{HLAT}}$. (e) AI-Powered Extraction confusion matrix evaluated on AI-Powered Extraction where only six entities are misclassified. (f) $\mathcal{D}_{\mathrm{HLAT}}$word cloud plots across all generated directions for all components (top), Auxiliary Info-Indication (bottom left), and Verb (bottom right). Larger size indicates higher frequency.

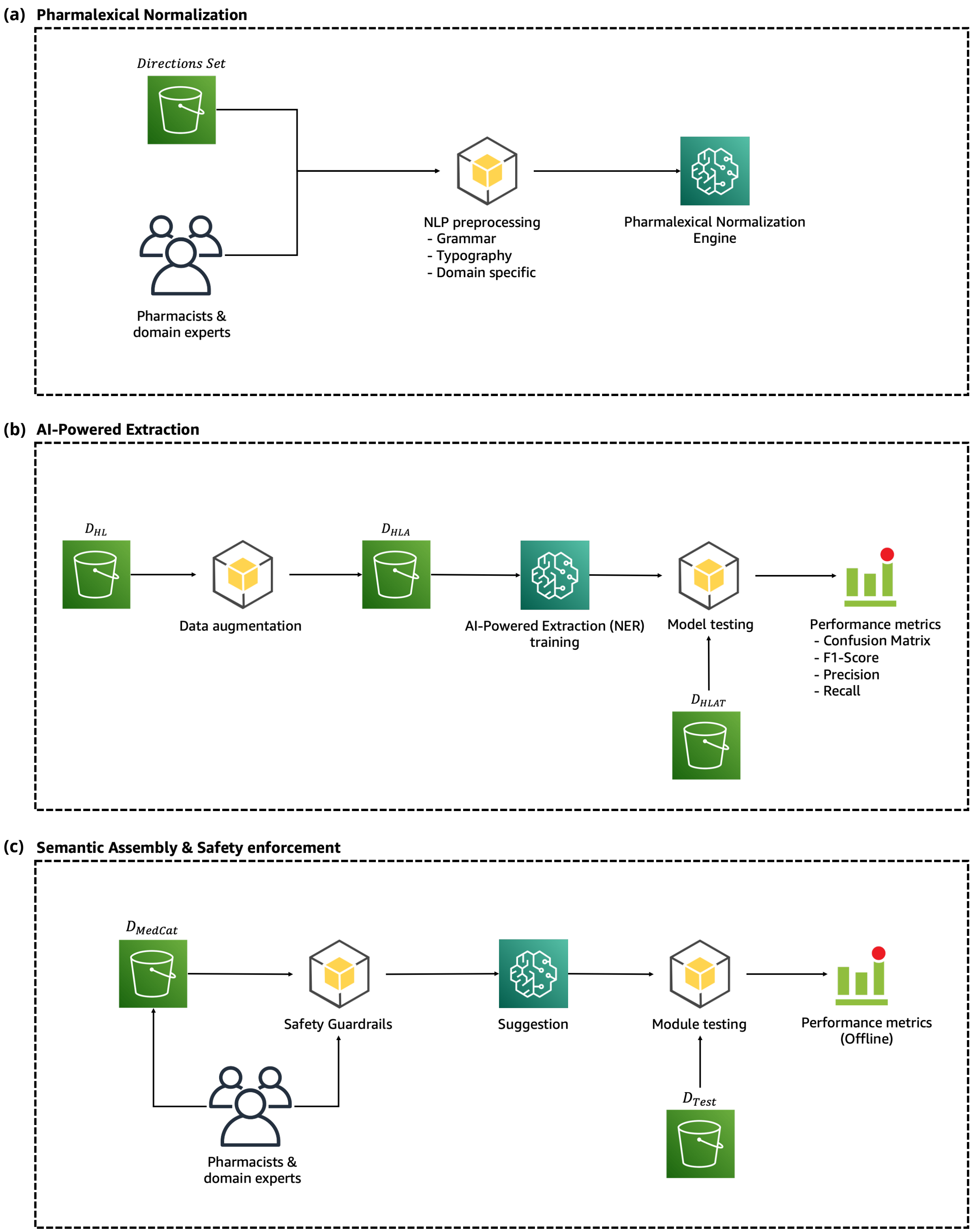

Supplementary Fig. 3. Data integration flow in the different stages of MEDIC. (a) Pharmalexical Normalization consolidates Directions Set with domain expert knowledge and traditional NLP normalization techniques to form a customize text normalization strategy specifically designed for medication directions. (b) AI-Powered Extraction central stage of MEDIC is trained using specifically designed data augmentation techniques applied to a human-labeled ($\mathcal{D}_{\mathrm{H}}$) dataset to detect all medically relevant components from the incoming directions, and tested on a similarly augmented ($\mathcal{D}_{\mathrm{HLAT}}$) set. (c) The final stage of MEDIC, Semantic Assembly & Safety Enforcement, employs $\mathcal{D}_{\mathrm{MedCat}}$ and pharmacy expertise to assemble the components extracted from AI-Powered Extraction and enforce safety guardrails on the final suggestions of MEDIC.

Supplementary Table 3. Examples highlighting the limitations of METEOR and BLEU to be used as the main performance metrics when patient safety plays a crucial role in the suggestion system. Low values for both metrics are defined as <0.7.

Ground Truth	Potential Suggestion	BLEU	METEOR
Take 1 tablet by mouth every day.	Take 1 and 1/2 tablet by mouth every day.	Low	High
Take 1 tablet by mouth every day.	Take 2 tablet by mouth every day.	Low	High
Dissolve 0.5 mg tablet under the tongue every day.	Dissolve 0.5 tablet under the tongue every day.	Low	High
Take 1 tablet by mouth once weekly.	Take 1 tablet by mouth once daily.	High	High
inhale 2 puffs orally every 6 hours. Stop after 10 days.	inhale 2 puffs orally every 4 hours. Stop after 10 days.	High	High
Apply lotion every day in the morning.	Apply lotion every day in the afternoon.	High	High

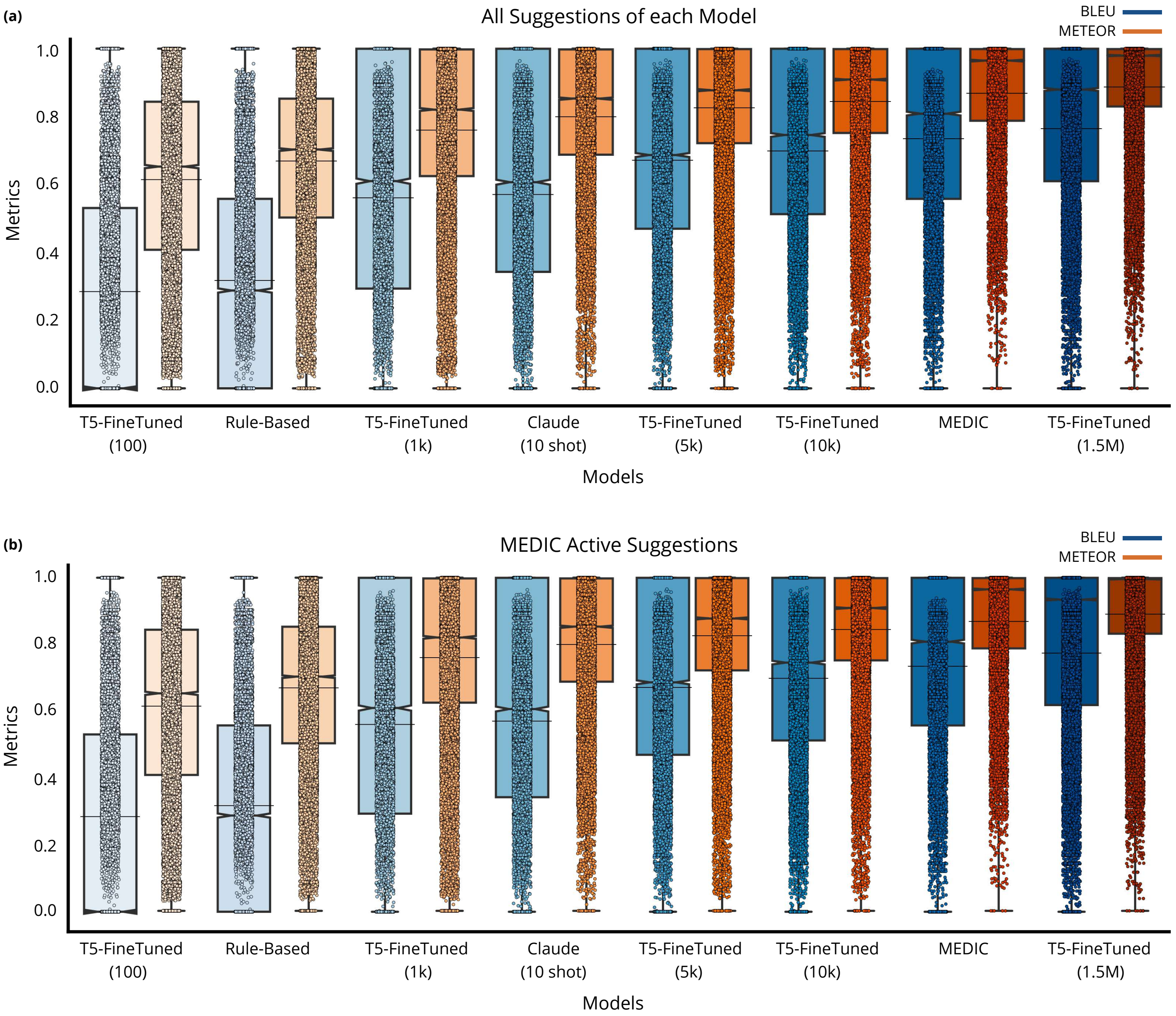

Supplementary Fig. 4. BLEU and METEOR results comparison. (a) Distribution of BLEU and METEOR metrics for the T5-FineTuned, MEDIC, Claude, and Rule-Based models, calculated across all suggestions of each model and (b) across all suggestions made by MEDIC. Average values are indicated with an horizontal black line and median values are highlighted with a notch on each boxplot from a total of n=1,200 prescriptions. Whiskers extend from the first and third quartiles (box limits) toward the min/max observed values for each metric and model, respectively. T5-FineTuned (100k) is omitted from the plot for visualization purposes (reaching performance between T5-FineTuned (10k) and MEDIC).
