# Supplementary material for: Large language models for preventing medication direction errors in online pharmacies
Source: Nat Med. 2024 Apr 25;30(6):1574–82. doi: 10.1038/s41591-024-02933-8 (PMC11186789; doi:10.1038/s41591-024-02933-8)
Supplement: Supplementary file 2 — Reporting Summary [file 41591_2024_2933_MOESM2_ESM.pdf]

nature portfolio

Corresponding author(s): Cristobal Pais

Last updated by author(s): Mar 8, 2024

Reporting Summary

Nature Portfolio wishes to improve the reproducibility of the work that we publish. This form provides structure for consistency and transparency in reporting. For further information on Nature Portfolio policies, see our Editorial Policies and the Editorial Policy Checklist.

Statistics

For all statistical analyses, confirm that the following items are present in the figure legend, table legend, main text, or Methods section.

n/a	Confirmed	
☐	☒	The exact sample size (*n*) for each experimental group/condition, given as a discrete number and unit of measurement
☐	☒	A statement on whether measurements were taken from distinct samples or whether the same sample was measured repeatedly
☒	☐	The statistical test(s) used AND whether they are one- or two-sided<br>*Only common tests should be described solely by name; describe more complex techniques in the Methods section.*
☒	☐	A description of all covariates tested
☒	☐	A description of any assumptions or corrections, such as tests of normality and adjustment for multiple comparisons
☐	☒	A full description of the statistical parameters including central tendency (e.g. means) or other basic estimates (e.g. regression coefficient) AND variation (e.g. standard deviation) or associated estimates of uncertainty (e.g. confidence intervals)
☐	☒	For null hypothesis testing, the test statistic (e.g. F, t, r) with confidence intervals, effect sizes, degrees of freedom and P value noted<br>*Give P values as exact values whenever suitable.*
☒	☐	For Bayesian analysis, information on the choice of priors and Markov chain Monte Carlo settings
☒	☐	For hierarchical and complex designs, identification of the appropriate level for tests and full reporting of outcomes
☒	☐	Estimates of effect sizes (e.g. Cohen's d, Pearson's r), indicating how they were calculated

Our web collection on statistics for biologists contains articles on many of the points above.

Software and code

Policy information about availability of computer code

Data collection	No software was used for data collection. Publicly available data was collected downloading it from their urls (see Data Availability statement). Internal data is owned/provided by Amazon Pharmacy.
Data analysis	Analysis code was written in Python (3.10.6).<br>The following list of Python packages were used: pandas: 1.4.4, numpy: 1.26.0, matplotlib: 3.5.2, seaborn: 0.11.2, Sagemaker: 2.100.0, Huggingface Transformers: 4.18.0, huggingface-hub: 0.8.1; Torch: 1.13.1+cu117, Re: 2.2.1; boto3: 1.24.34; botocore: 1.27.59; compress-pickle 2.1.0; nltk: 3.8; wordcloud: 1.8.2.2

For manuscripts utilizing custom algorithms or software that are central to the research but not yet described in published literature, software must be made available to editors and reviewers. We strongly encourage code deposition in a community repository (e.g. GitHub). See the Nature Portfolio guidelines for submitting code & software for further information.

Data

Policy information about availability of data

All manuscripts must include a data availability statement. This statement should provide the following information, where applicable:

- Accession codes, unique identifiers, or web links for publicly available datasets
- A description of any restrictions on data availability
- For clinical datasets or third party data, please ensure that the statement adheres to our policy

The RxNorm and FDA datasets are publicly available from the National Library of Medicine website and U.S. Food and Drug Administration openFDA website, respectively (https://www.nlm.nih.gov/research/umls/rxnorm/index.html and https://open.fda.gov/).
The remaining datasets generated during the study cannot be made publicly available due to HIPAA and Amazon LLC policies. Please, contact Amazon Science services (amazonscience-website@amazon.com) for inquiries about the proprietary datasets used in this study.

Extra information:
For each medication direction satisfying the inclusion criteria, we extract data from the Amazon Pharmacy Database. Each sample is characterized by the following fields: i) id: identifier of a unique medication direction; ii) drug id: an internal identifier of the drug; iii) directions: raw digital directions from prescribers; and iv) typed directions: archived prescriptions typed by pharmacy technicians and verified by pharmacists. Additionally, RxNorm from National Library of Medicine and OpenFDA National Drug Code Directory were used, leveraging their National Drug Codes (NDCs) to obtain medication description for all Amazon Pharmacy medications. More details are provided in Section 1.2 of the paper.

Human research participants

Policy information about studies involving human research participants and Sex and Gender in Research.

Reporting on sex and gender	No data on gender was collected
Population characteristics	No data on race, ethnicity, or other social relevant groupings were collected. We only had access to the medication directions and the medication information, for each prescription
Recruitment	No such data was collected nor was available.
Ethics oversight	Amazon Scientific Publishing and Amazon Legal

Note that full information on the approval of the study protocol must also be provided in the manuscript.

Field-specific reporting

Please select the one below that is the best fit for your research. If you are not sure, read the appropriate sections before making your selection.

☒ Life sciences ☐ Behavioural & social sciences ☐ Ecological, evolutionary & environmental sciences

For a reference copy of the document with all sections, see nature.com/documents/nr-reporting-summary-flat.pdf

Life sciences study design

All studies must disclose on these points even when the disclosure is negative.

Sample size	A random subsample of medication directions data was extracted from a year's worth of historical single-line directions from Amazon Pharmacy. This subset (nearly 1.6 million prescriptions) underwent formatting and cleaning processes to remove non-valid prescriptions, resulting in a representative dataset suitable for training and quantitative evaluation against alternative benchmarks.<br><br>The sample size for labeling data was limited to 1,000 cases due to cost constraints associated with labor annotation. However, we ensured representativeness by stratified randomization, ensuring that each dataset contained a diverse sample of different types of directions. This stratification process was guided by clustering a lower-dimensional representation of all distinct raw directions found in the dataset.
Data exclusions	Non-valid prescriptions, such as those lacking medication directions or drug information (less than 0.1% of all cases).<br>We also excluded medication directions involving multi-line directions (<2% of all cases).
Replication	The experiments involving training ML models on real and synthetic data, as well as testing on real datasets, were replicated independently for each prescription. Additionally, the main model MEDIC underwent testing in a production environment. Throughout the process, the models' performance remained consistent, and detailed outputs were thoroughly analyzed using widely-used NLP metrics and expert manual reviews. All attempts at replication were successful, yielding reliable and consistent results across experiments. Each measurement was repeated n>=10 times

Randomization	The initial 1.6M samples were selected randomly from one year of prescription data at Amazon. All remaining sub-setting of this data is performed randomly as stated in Section 1.2.
Blinding	This study contains no control or placebo arm and therefore blinding is not applicable

Reporting for specific materials, systems and methods

We require information from authors about some types of materials, experimental systems and methods used in many studies. Here, indicate whether each material, system or method listed is relevant to your study. If you are not sure if a list item applies to your research, read the appropriate section before selecting a response.

Materials & experimental systems

n/a	Involved in the study
☒	☐ Antibodies
☒	☐ Eukaryotic cell lines
☒	☐ Palaeontology and archaeology
☒	☐ Animals and other organisms
☐	☒ Clinical data
☒	☐ Dual use research of concern

Methods

n/a	Involved in the study
☒	☐ ChIP-seq
☒	☐ Flow cytometry
☒	☐ MRI-based neuroimaging

Clinical data

Policy information about clinical studies

All manuscripts should comply with the ICMJE guidelines for publication of clinical research and a completed CONSORT checklist must be included with all submissions.

Clinical trial registration	No clinical trial or randomized controlled study was performed. All of the analysis was on retrospective data except one part which was testing of our MEDIC method in Amazon Pharmacy production system. This was approved after the method demonstrated in all retrospective tests that it would improve quality of prescription processing, in line with quality improvement initiatives, it was tested in the Pharmacy production system, as a before and after study.
Study protocol	This study was reviewed and approved by relevant IRB entities at Amazon as well as Amazon Scientific Publishing
Data collection	No clinical trial or randomized controlled study was performed.
Outcomes	No clinical trial or randomized controlled study was performed.
